# Validation of the European Drug Addiction Prevention Trial Questionnaire (EU-Dap) for substance use screening and to assess risk and protective factors among early adolescents in Chile

**DOI:** 10.1371/journal.pone.0258288

**Published:** 2021-10-11

**Authors:** Saray Ramírez, Sofía Gana, María Inés Godoy, Daniela Valenzuela, Ricardo Araya, Jorge Gaete

**Affiliations:** 1 Research Center for Students Mental Health (ISME), Faculty of Education, Universidad de los Andes, Santiago, Chile; 2 Department of Educational Assessment, Measurement and Registry, Universidad de Chile, Santiago, Chile; 3 Department of Psychology INVEST Research Flagship, University of Turku, Turku, Finland; 4 ANID, Millennium Science Initiative Program, Millennium Nucleus to Improve the Mental Health of Adolescents and Youths, Imhay, Santiago, Chile; 5 Department of Health Service & Population Research, King’s College London, London, United Kingdom, David Goldberg Centre, Denmark Hill, London, United Kingdom; Universidade Federal de Sao Paulo, BRAZIL

## Abstract

**Background:**

Substance use is highly prevalent among Chilean adolescents, and the damage it causes at the neurobiological, psychological, and social levels is known. However, there are no validated screening instruments that also assess risk and protective factors for this population in Chile, which is essential for evaluating future prevention interventions.

**Objective:**

To determine the psychometric properties of the European Drug Addiction Prevention Trial Questionnaire (EU-Dap) questionnaire.

**Methods:**

A cross-sectional study was carried out in 13 schools in the city of Santiago de Chile. The sample included 2261 adolescents ranging from 10 to 14 years old. Linguistic and cultural adaptation was assessed using focus groups with adolescents, the construct validity was evaluated using confirmatory factor analysis, and measures of its reliability were also determined. Furthermore, the associations regarding risk and protective factors with substance use were explored.

**Results:**

Substance use questions were well understood and seemed to adequately capture the consumption of different drugs. Regarding the subscales of risk and protective factors, the analyses showed that most subscales had good psychometric properties, and few needed some degree of improvement (e.g., some items were removed). After the changes, most final subscales had good or adequate goodness of fit adjustments and good or acceptable internal consistency. Finally, the main associated factors with the substance use outcomes were: future substance use and school bonding for tobacco use; negative beliefs about alcohol, future substance use, school bonding and refusal skills for alcohol use; and negative beliefs about marihuana, positive attitudes towards drugs, risk perception, and substance abuse index for marihuana use. Normative beliefs increased the risk for all substances use.

**Conclusions:**

The current findings suggest that the EU-Dap is a valid and reliable instrument, and it may help to evaluate the effectiveness of drug use prevention interventions.

## Introduction

Most adults with substance abuse disorders started substance use during adolescence, and it is known that the earlier life consumption begins, the greater the risk of dependence in the future [1, 2]. This is partly because crucial brain development changes that affect cognitive, behavioral, and mood functions occur during adolescence [3–6].

Substance use and mental disorders are among the main contributors to disease among children and adolescents in the Americas region, representing 5.2% of the disability-adjusted life years (DALYs) and 17.2% of the years lived with a disability (YLD) in the population from 0 to 14 years.

In 2015, Chile led this region in tobacco use in the last month among adolescents in secondary education, reaching a prevalence of 23.7% [7]. Regarding the prevalence of marijuana use in the previous year among adolescents in secondary education, Chile also led with 32.8% [7]. It should be noted that this last indicator has doubled over the previous two decades from 14.8% in 2001 to 30.9% in 2017 [8]. Regarding the prevalence of alcohol use in the last month among secondary school adolescents, it has remained relatively stable in the previous two decades, being 38.9% in 2011 and 31.1% in 2017 [8].

Substance use among adolescents has been associated with the onset of psychiatric disorders such as depression and anxiety disorders [9, 10], in addition to dependency and substance abuse [10, 11]. That is why in Chile, since 2017, the state has legislated the Explicit Health Guarantee (GES) for the Harmful Use and Dependence of Alcohol and Other Drugs for people under 20 years old. The law grants a set of benefits for the treatment and follow-up of alcohol and drug abuse, and it has covered a total of 24,589 cases up to December 2019 [12].

Overall, substance use has a significant economic impact in Chile. For example, in 2019, the costs of only the GES benefits were $319,714,800. In addition, approximately $899,388 is spent annually on hospitalization for alcohol and drug detoxification in adolescents. This figure is estimated using the data for the average cost per day of hospitalization, which is $45 in the year 2020 [13]; the average number of days of stay for mental and behavioral disorders due to the use of psychoactive substances, which is 17.2 days in the year 2017; and a total of 1162 discharges of adolescents ranging from 10 to 19 years of age in that same year [14].

In order to prevent substance use and its consequences, several school-based programs have been evaluated worldwide [15]. For instance, programs such as “Life Skills Training”, “Project Towards No Drug Abuse (TND)”, and “All Stars” can delay or prevent adolescent tobacco, alcohol, and cannabis use [15, 16]. In particular, one of the most known preventive programs is the Unplugged program, which has been shown to be effective in seven European countries [17–19] and highlighted as one of the best preventive programs in the world in recent systematic reviews [16, 20].

To assess the prevalence of substance misuse, screen this problem among adolescents, and evaluate the effectiveness of preventive interventions [20], it is necessary to have validated and reliable instruments. Worldwide, several questionnaires have been used and validated for these purposes, such as “The Car, Relax, Alone, Forget, Family/friends, and Trouble questionnaire” (CRAFFT) [21], “Alcohol Use Disorders Identification Test” (AUDIT) [22], and The Cannabis Use Problems Identification Test (CUPIT) [23]. However, most of these instruments have been used for specific substance use (e.g., AUDIT for alcohol use; and CUPIT for cannabis use) and have not been validated in a population of early adolescents, particularly in Chile. For instance, one Chilean study [24] conducted the validation of the AUDIT, created and disseminated by the World Health Organization (WHO) [22]. The study reported satisfactory psychometric properties; however, it did not include early adolescents [24]. On the other hand, we can mention the Chilean governmental efforts performed by the National Service for the Prevention and Rehabilitation of Drugs and Alcohol (SENDA), which has implemented its questionnaire every two years since 1995 in the “National Study of School Population in Chile”. Unfortunately, there are no validation studies of this instrument or evaluation of the effectiveness of government programs using this questionnaire.

Other instruments have been associated with the evaluation of prevention programs. Two of the most effective programs, the LifeSkills Training program [15] and the Unplugged program [16], have used their own questionnaires to assess the effectiveness of the interventions. Regarding Unplugged, this program has a self-reported instrument called the “European Drug Addiction Prevention Trial Questionnaire” (EU-Dap), which evaluates substance use and several risk and protective factors among adolescents. The intended structure of this questionnaire includes a total of 16 subscales (personal communication with authors), measuring risk or protective factors for substance use among adolescents. The assessment of risk and protective factors allows exploring the mediating factors of the effectiveness of an intervention. For instance, in Europe, it was found that normative beliefs, positive attitudes towards drugs, and refusal skills mediated the intervention effects on tobacco and alcohol use among adolescents [25]. The EU-Dap questionnaire has been widely used in Europe [26], and there is only one validation study, conducted in Brazil [27], where the Unplugged prevention program (“#Tamojunto”) has also been evaluated [28]. This validation was mainly focused on translation and cultural adaptation, with no exploration of its structure of items. Additionally, they had high nonresponse rates, especially in the last questions of the questionnaire, reaching up to 47% of missing data, and low consistency of the questions on drug use (Kappa: 0.40–0.59) and risk and protective factors (Kappa: 0.20–0.39). In sum, no previous studies have assessed the internal structure of the scales included in the EU-Dap questionnaire.

Overall, it is crucial to have validated instruments to assess preventive interventions. Additionally, the validation process must be performed in a sample with similar features regarding age and cultural context to the population where the intervention is planned to be implemented [29, 30].

We are conducting a large study to assess the effectiveness of the culturally adapted Unplugged program in Chile (known as “Yo Sé Lo Que Quiero” [“I Know What I Want”]). In order to obtain robust results from this evaluation, we need to have a validated instrument; therefore, we decided to use the same questionnaire as in Europe and Brazil to perform future comparisons between countries. Thus, the aims of this study were the following: (1) culturally adapt the EU-Dap questionnaire to early adolescents in Chile, (2) assess the validity of the internal factor structure of the subscales contained in the questionnaire evaluating risk and protective factors, (3) assess the reliability of these subscales and (4) assess the possible relationships between all risk and protective factors and substance abuse measured by the questionnaire.

## Materials and methods

### Study design

Two studies were conducted in this validation. Study 1 refers to the linguistic and cultural adaptation of the questionnaire and a pilot study of the understanding of the questions in a small group of adolescents attending 5^th^ to 8^th^ grades. Study 2 refers to a cross-sectional study conducted in a large sample of students attending the same grades, aiming to assess the psychometric properties of the instrument.

### Study 1

#### Linguistic and cultural adaptation of the EU-Dap questionnaire

For the study, a cultural adaptation was made based on the Spanish version of the EU-Dap questionnaire, where Spanish idioms and words that are not used in Chile were changed. Additionally, the English version was used along with the Spanish version to better understand the meaning of each of the questions to avoid potential cultural adaptations present in the Spanish version used in Spain. It is worth mentioning that the original author of the EU-Dap questionnaire gave us permission to use and modify the original questionnaire in the context of this cultural adaptation.

The resulting questionnaire was piloted in one class per level (the total number of students was 140, including 36 5^th^ grade students, 36 6^th^ grade students, 33 7^th^ grade students, and 35 8^th^ grade students), from one school representing the middle-income status, and it was selected for convenience. Later, two focus groups were subsequently held inviting students who previously answered the questionnaire, as described above: the first focus group included 5^th^ and 6^th^ graders and the second, 7^th^ and 8^th^ graders. Regarding the selection of the participants in each focus group, six students per class were randomly selected (a total of 12 students participated in each focus group); and there were equal proportions of males and females. The purpose of these focus groups was to assess in-depth the understanding and wording of the questions included in the questionnaire. The qualitative data analysis of the focus groups was evaluated by content analysis and Grounded Theory [31].

### Study 2

#### Setting and sample

This study is part of a large research project aiming to assess the effectiveness of the “Yo Sé Lo Que Quiero” (Unplugged) in Chile through a cluster randomized controlled trial (cRCT), registered at ClinicalTrials.gov, under the identifier NCT04236999. The program will be implemented among 6th and 7th graders. In this cRCT, the baseline assessment will be carried out when the students are attending 5th and 6^th^ grades; then when the intervention is completed (that is, students will be in 6^th^ and 7^th^ grades); and, finally, 12 months after the intervention (that is, when students will be in 7th and 8th grades). For this reason, the questionnaire validation was designed to evaluate the psychometric properties of the instrument for the entire age group that could answer the questionnaire during the cRCT, that is, 5^th^ to 8^th^ grade students (ages 10–14). It is worth mentioning that the enrolment process of the validation study is completely independent of the cRCT. Therefore, the selected schools for each study will be different.

#### Sample size

Different criteria have been suggested to calculate the sample size for this kind of study [32]. A rule of thumb has been considered that the minimum number of subjects per item should be between 5 and 20 subjects [33]. We decided to use the middle point of this range (12.5:1). Since the questionnaire to be validated had a total of 174 items, at least 2,175 subjects would be required. Due to a potential response rate of 50–60% in this kind of investigation [34], we expected to recruit between 13 and 14 schools, considering an average of two classes per grade and 30 students per class. This resulted in a potential sample size between 3,120 and 3,360. For practical reasons, such as access to schools and prior relationship between schools and research team, the sampling procedure chosen was by convenience. To estimate the statistical power of the resulting sample size, the calculation of the effect size of the association between refusal skills (one of the independent variables included in the questionnaire) and alcohol use in the last 30 days (dependent variable) was performed.

#### Recruitment

The educational system in Chile is structured into three types of primary and secondary schools: 1) public fully state-funded schools (44.6% of students attend these schools); 2) subsidized schools, which are administered by private nonprofit organizations, which also receive state funds (49.5%); 3) and private schools, which are administered by private organizations (either nonprofit or for-profit), and they do not receive state funds (5.9%) [35]. Regarding school composition in Chile, schools may be single-sex (3.9%) or mixed-sex (96,1%) [36].

The eligible schools for this validation study were all located in Santiago, Chile. Inclusion criteria were (1) mixed-sex schools and (2) schools that represented high, medium, or low socioeconomic levels according to the categorization reported by the Educational.

Quality Agency [37]. This categorization considers the following socioeconomic variables to group the schools: mother’s educational level, father’s educational level, total monthly household income, and vulnerability index [37]. The latter index is built every year by the Ministry of Education and assigned to every school based on poverty conditions and the risk of school failure of the students [34]. From all schools contacted, 13 consented to participate. Of the participating schools, seven came from low socioeconomic levels with a total enrollment of 920 students from 5th to 8th grade, four came from middle socioeconomic levels with a total of 1,103 students, and two came from high socioeconomic levels with a total of 1,004 students. Therefore, a total of 3,027 students were eligible to participate and were invited. Of these, a total of 2,261 (74.7%) consented and responded to the questionnaire.

#### Procedure and ethical considerations

All evaluation data were collected following the Declaration of Helsinki with the approval of the ethics committee of the Universidad de los Andes (CEC201734, August 7th, 2018). Participation in the study involved three stages: First, school authorities were informed about the study, and written confirmation was requested to participate. Then, the parents were sent a letter with the study information and a form requiring written and informed consent. Finally, the students were informed about the study and asked to sign an agreement confirming their participation. Confidentiality and the freedom to withdraw from it at any time were assured throughout the study. Anonymous codes were generated to protect the identities of the participants. The data were collected from August to December 2018 by research assistants trained by the study coordinator. During the questionnaire application, the research assistants explained the objectives of the study, clarified the doubts of the students, and then asked for their agreement. No teachers from the schools were involved during the assessment.

#### Data analysis

Descriptive analysis was performed, and relative frequencies and percentages were calculated by gender, family structure, socioeconomic level, and administrative dependency variables. Tobacco, alcohol, and marijuana use prevalence were reported in the following periods: last 30 days, last 12 months, and lifetime. The prevalence of other substances, such as hallucinogens or cocaine, was not reported because of the negligible number of students who used these drugs. Regarding the EU-Dap subscales, means, standard deviations, kurtoses, and skewnesses were calculated.

Additionally, the psychometric properties of the EU-Dap questionnaire were addressed in the following order.

#### Evidence of the internal structure of the EU-Dap questionnaire

The dimensionality, reliability, and structure of the original 16 subscales of the EU-Dap instrument were studied in several steps: (1) Polychoric correlation matrices, with a distribution of skewness and kurtosis coefficients, were calculated for each scale. The factor loadings were calculated, and the commonality of items was analyzed according to the following saturations: >0.70 was considered optimal, 0.40–0.70 was considered moderate, and 0.30–0.39 was considered minimal [38]. (2) The degree of adequacy of the sampling of the polychoric matrix for exploratory factorization was verified with the Kaiser-Meyer-Olkin index (KMO) under the measure of sampling adequacy (MSA), which indicates that a value equal to or above 0.5 is acceptable to perform factor analysis [39]. (3) The factors were estimated with the unweighted least squares method (ULS), through exploratory factor analysis with no rotation that revealed the internal structure of the covariance or correlation matrices [40]. (4) The number of suitable common factors was selected using parallel analysis, which presents eigenvalues greater than those that would be obtained by chance [41]. (5) Additionally, if exploratory factor analysis showed more of one dimension for a subscale, a Promax rotation method was used, and factor loading was updated accordingly. The Promax method is one of the oblique methods recommended when factors are correlated [42].

Finally, (6) the reliability was evaluated using the omega coefficient, where values of 0.65 or more are considered acceptable [43].

Given the evidence of the internal structure of the EU-Dap instrument, we decided either to keep or to remove some items from the original subscales according to the following two criteria: (1) The allocation of the items to each of the factors was considered acceptable if the saturation (factor loading) was over 0.40 [38]; (2) Items that loaded in different scales or factors (cross-loading), with factor loading over 0.40, was kept in the factor with the highest saturation (see Table 3 and Table 4).

#### Evidence of the internal structure of the final subscales of the EU-Dap questionnaire

Confirmatory factor analysis (CFA) was performed using the information obtained from the exploratory factor analyses. When one factor was revealed, a model of one factor was conducted in the CFA. When two factors were shown, a 2-correlated model was performed in the CFA [32]. The goodness of fit of the proposed models was evaluated using the following parameters recommended by Schermelleh-Engel and colleagues [44]: (1) the root mean square error of approximation (RMSEA), where values above or equal to 0 and below or equal to 0.05 were considered to be a good fit and values between 0.06 and 0.08 were considered to be an acceptable fit; (2) the standardized mean square residual root (SRMR), where values above or equal to 0 and below or equal to 0.05 were considered to be a good fit, and values between 0.06 and 0.10 were considered to be an acceptable fit; and (3) the comparative adjustment index (CFI), where values above or equal to 0.97 and below or equal to 1.00 were considered to be a good adjustment, and values between 0.95 and 0.96 were considered to be an acceptable fit.

#### Associations of EU-Dap risk and protective factors and substance use

The association analyses aimed to explore all potential risk and protective factors for several substance use outcomes using univariable models and determine which of the EU-Dap subscales were significantly associated with substance use outcomes using multivariable models.

First, we explored univariable associations. The independent variables were all EU-Dap subscales, the items that did not enter in any of the scales, and other items included in the questionnaire playing a role as risk and protective according to the theoretical model of the “Yo Sé Lo Que Quiero” (Unplugged) program [45]. In the case of positive and negative beliefs about tobacco, alcohol, and marijuana use scales, they were considered as independent variables only for the correspondent substance use (e.g., positive beliefs about tobacco on the 30-day prevalence of tobacco). Additionally, positive and negative attitudes towards drugs were only included in the analyses regarding marihuana use because these two scales only explored attitudes towards illegal substances. Dependent variables were the 30-day prevalence of tobacco, alcohol, and marijuana.

Additionally, we included other valuable outcome measures of substance use, such as the 30-day prevalence of drunkenness, the 30-day prevalence of binge drinking (males and females), and the 12-month prevalence of marijuana use. In the case of binge drinking, we used the consensus definition by the literature, where the number of drinks was different for males (5 or more on the same occasion) and females (4 or more on the same occasion) [46]. All dependent variables were analyzed as dichotomous variables (yes/no), and performing logistic regression models, accordingly. The odds ratios were examined with 95% confidence intervals, and the cutoff for statistical significance was established with a p-value < 0.05.

Second, multivariable associations were performed only for the EU-Dap subscales. These logistic regression models included all subscales associated with the different outcomes. Additionally, each logistic regression multivariable model was adjusted by gender and age. The odds ratios were examined with 95% confidence intervals, and the cutoff for statistical significance was established with a p-value < 0.05.

Internal structure statistical analyses were conducted using R 3.5.0 software, and CFA was performed using the lavaan package in R 3.5.0. Finally, descriptive and associations analyses were conducted using Stata 15.

### Measures

#### EU-Dap questionnaire

The EU-Dap has 45 questions and collects information on substance use, knowledge, attitudes, and opinions about alcohol, tobacco, and other drug use. Regarding the structure of the answers, most questions use Likert-type scale response anchors, but are different for different questions. For each scale the answers are summed up. See S1 and S2 Questionnaires.

It consists of the following sections:

1. Gender, age, and family structure (no. of items = 13): Data on gender (1 = male and 2 = female) and age were collected, along with family structure (1 = Lives with father, 2 = Lives with mother, and 3 = Lives with siblings). Other variables regarding the identification of other family members can be found in S1 Table.
2. Substance use prevalence, which includes the following: tobacco use (no. of items = 4), alcohol use (no. of items = 10), marijuana use (no. of items = 3), and use of other drugs (no. of items = 8).
3. Risk and Protective factors subscales, which include the following: positive and negative beliefs about tobacco use (no. of items = 8), positive and negative beliefs about alcohol use (no. of items = 10), positive and negative beliefs about marijuana use (no. of items = 10), future substance use (no. of items = 6), positive and negative attitudes towards drugs or illegal substances (no. of items = 11), risk perception (no. of items = 8), normative beliefs (no. of items = 5), parental involvement (no. of items = 5), family functioning (no. of items = 20), school bonding (no. of items = 5), substance abuse index (no. of items = 11; including problems attributed to alcohol or drug use; for example, Have you ever had any accidents or injuries in the last 12 months due to alcohol or drug use?), decision-making skills (n. of items = 5), refusal skills (no. of items = 3), self-esteem (no. of items = 10), poor problem-solving skills (no. of items = 5) and assertiveness (no. of items = 6). For more details, see S2 Table.
4. Other risk and protective factors, which include the following: knowledge about substances (no. of items = 6), tobacco use by family and friends (no. of items = 4), alcohol and drug use by siblings (no. of items = 5), parental permissiveness (no. of items = 6), self-reported school performance (no. of items = 1), and positive academic expectations (no. of items = 1). For more details, see S3 Table.

## Results

### Study 1

#### Linguistic and cultural adaptation of the EU-Dap questionnaire

Students from focus groups had a positive general opinion of the content and layout of the questionnaire. The format of the questions regarding substance use was well received, and students valued the questions regarding the risk and protective factors. They considered that most of the questions were appropriate and had a good understanding. There was no particular difficulty of understanding for younger (5^th^ and 6^th^ graders) than older students (7^th^ and 8^th^ graders); therefore, it was not necessary to change the content and format between populations. Some students raised a concern regarding the questions about family structure, which were considered too personal. However, in this and all procedures involving data collection, it was assured that the data would be managed with high standards of confidentiality. Finally, students agreed to keep the questions in the questionnaire without further concerns.

Regarding the understanding of the questions, only six of them required some changes. For example, the original questionnaire had a 3X3 table for tobacco, alcohol, and marijuana use and the three periods assessed regarding the prevalence (30 days, 12 months, and lifetime). Still, the students considered this table to be too complicated to understand and answer. Therefore, we decided to use different questions for each substance and each period. Another example was present in the subscale called “positive and negative attitudes towards drugs”, where there were some problems with the meaning of some of the expressions used in the original questionnaire. One question stated that “drug use can be a pleasurable activity”, but the students did not clearly understand the translation to Spanish; therefore, we changed this item to the statement “drug use can be an enjoyable activity”, based on the meaning of the English version of the questionnaire. Another question stated, “drugs help people to have a fulfilling life experience”, which also had problems being understood; therefore, we changed it for “drugs help people to have a completely happy life”. Finally, there was one question on the family dynamics subscale that stated, “In my family, we rarely lose our temper (we get out of hand)”, which had problems being understood; therefore, we changed it to “In my family, we rarely lose control”.

### Study 2

#### Description of the sociodemographic variables

A total of 2,261 students participated. Their ages ranged from 10 to 14 years old, and 53,3% [51.3–55.4] were male. Most students live with their parents and siblings. The average age for each grade level was 10.7 (SD = 0.65) years old for 5^th^ grade, 11.8 (SD = 0.72) years old for 6^th^ grade, 12.8 (SD = 0.73) years old for 7^th^ grade, and 13.8 (SD = 0.74) years old for 8^th^ grade. The students attended schools with different administrative dependencies: 38.9% [36.9–40.9] attended a public school, 28.9% [27.1–30.8] attended a subsidized school, and 32.2% [30.3–34.2] attended a private school. Students came from different school socioeconomic backgrounds in equal proportions: 30.5% [28.7–32.4] came from a low socioeconomic level, 37.3% [35.3–39.3] came from a medium socioeconomic status, and 32.2% [30.3–34.2] came from a high socioeconomic level. See Table 1.

**Table 1:** Sociodemographic variables.

Variables	n	% or Mean	[95% CI] or (SD)
Gender			
Female	1055	46.7	[44.6–48.7]
Male	1206	53.3	[51.3–55.4]
Family Structure			
Lives with father	1416	69.6	[67.5–71.5]
Lives with mother	2049	94.0	[92.9–94.9]
Lives with siblings	1831	87.3	[85.8–88.6]
Socioeconomic Level			
High	728	32.2	[30.3–34.2]
Medium	843	37.3	[35.3–39.3]
Low	690	30.5	[28.7–32.4]
Type of School dependency			
Private	728	32.2	[30.3–34.2]
Subsidized	654	28.9	[27.1–30.8]
Public	879	38.9	[36.9–40.9]
Class grade			
5^th^	539	23.8	[22.1–25.6]
6^th^	615	27.2	[25.4–29.1]
7^th^	564	24.9	[23.2–26.8]
8^th^	543	24.0	[22.3–25.8]
Age by Class grade			
5^th^	522	10.7	(0.65)
6^th^	611	11.8	(0.72)
7^th^	562	12.8	(0.73)
8^th^	536	13.8	(0.74)

#### Substance use prevalence

Regarding tobacco use, there is an increase with age for every period measured, and it is more prevalent among females in the 8^th^ grade. For example, the last month prevalence among 8^th^ graders was 5.9% [3.5–9.7] for females and 3.8% [2.1–6.8] for males. The prevalence of alcohol use was the highest compared to the other substances, with the lifetime prevalence reaching 39.2% [37.2–41.3] among all grades. Males passed females regarding alcohol use in all periods for 5^th^-7^th^ graders; however, among 8^th^ graders, females had a higher prevalence in all periods. 2.1% [1.6–2.8] had been drunk in the last 30 days, and the highest proportion was among 8^th^ graders. In every grade, the prevalence of binge drinking in the last 30 days was higher among females than males, especially among 8^th^ graders (females, 12.1% [8.5–16.9]; and males, 4.8% [2.9–8.0]). Like alcohol use, males passed females regarding marijuana use in all periods for 5^th^-7^th^ graders; however, among 8^th^ graders, females had a higher prevalence in all periods. (Table 2). Finally, the percentage of the missing values for each question of prevalence ranged between 0.9 and 2.7.

**Table 2:** Substance use variables.

Variables		Total		Females		Males	
Tobacco use in the last month	Grade	n	% [95% CI]	n	% [95% CI]	n	% [95% CI]
	5^th^	7	1.3 [0.6–2.7]	4	1.6 [0.6–4.1]	3	1.1 [0.3–3.3]
	6^th^	27	4.4 [3.0–6.3]	13	4.5 [2.6–7.6]]	14	4.3 [2.6–7.1]
	7^th^	16	2.8 [1.8–4.6]	8	3.1 [1.5–6.0]	8	2.6 [1.3–5.2]
	8^th^	25	4.7 [3.2–6.9]	14	5.9 [3.5–9.7]	11	3.8 [2.1–6.8]
Total		75	3.4 [2.7–4.2]	39	3.7 [2.7–5.1]	36	3.0 [2.2–4.2]
Total missing values		25	1.1 [0.7–1.6]	11	1.0 [0.6–1.9]	14	1.2 [0.7–2.0]
Tobacco use in the last year	5^th^	12	2.3 [1.3–3.9]	5	1.9 [0.8–4.6]	7	2.5 [1.2–5.2]
	6^th^	27	4.4 [3.1–6.4]	9	3.2 [1.6–6.0]	18	5.6 [3.5–8.7]
	7^th^	30	5.4 [3.8–7.6]	13	5.0 [2.9–8.5]	17	5.6 [3.5–8.9]
	8^th^	72	13.7 [11.0–16.9]	43	17.9 [13.6–23.3]	29	10.1 [7.1–14.2]
Total		141	6.3 [5.4–7.4]	70	6.7 [5.4–8.4]	71	6.0 [4.8–7.5]
Total missing values		32	1.4 [1.0–2.0]	14	1.3 [0.8–2.2]	18	1.5 [0.9–2.4]
Lifetime tobacco use	5^th^	28	5.3 [3.7–7.5]	14	5.5 [3.3–9.1]	14	5.1 [3.0–8.4]
	6^th^	46	7.5 [5.7–9.9]	15	5.2 [3.2–8.5]	31	9.5 [6.8–13.3]
	7^th^	60	10.7 [8.4–13.5]	29	11.2 [7.9–15.6]	31	10.3 [7.3–14.2]
	8^th^	115	21.7 [18.4–25.5]	65	27.1 [21.8–33.1]	50	17.3 [13.4–22.1]
Total		249	11.2 [9.9–12.5]	123	11.8 [10.0–13.9]	126	10.6 [9.0–12.5]
Total missing values		29	1.3 [0.9–1.8]	14	1.3 [0.8–2.2]	15	1.2 [0.8–2.1]
Alcohol use in the last 30 days	5^th^	22	4.1 [2.7–6.2]	9	3.5 [1.8–6.6]	13	4.7 [2.7–7.9]
	6^th^	31	5.1 [3.6–7.1]	13	4.5 [2.6–7.7]	18	5.5 [3.5–8.6]
	7^th^	62	11.1 [8.7–14.0]	25	9.7 [6.6–13.9]	37	12.3 [9.1–16.6]
	8^th^	105	19.8 [16.7–23.5]	52	21.8 [17.0–27.5]	53	18.3 [14.2–23.2]
Total		220	9.8 [8.7–11.2]	99	9.5 [7.9–11.4]	121	10.2 [8.6–12.0]
Total missing values		27	1.2 [0.8–1.7]	13	1.2 [0.7–2.1]	14	1.2 [0.7–2.0]
Alcohol use in the last year	5^th^	64	12.0 [9.5–15.0]	27	10.5 [7.3–14.9]	37	13.4 [9.8–17.9]
	6^th^	76	12.4 [10.0–15.3]	30	10.5 [7.4–14.6]	46	14.2 [10.8–18.4]
	7^th^	149	26.6 [23.1–30.4]	63	24.4 [19.5–30.0]	86	28.5 [23.7–33.8]
	8^th^	216	41.1 [36.9–45.3]	107	45.0 [38.7–51.3]	109	37.8 [32.4–43.6]
Total		505	22.6 [20.9–24.4]	227	21.8 [19.4–24.5]	278	23.3 [21.0–25.8]
Total missing values		30	1.3 [0.9–1.9]	16	1.5 [0.9–2.5]	14	1.2 [0.7–2.0]
Lifetime alcohol use	5^th^	136	25.7 [22.1–29.6]	58	22.7 [18.0–28.3]	78	28.4 [23.3–34.0]
	6^th^	197	32.2 [28.6–36.1]	78	27.3 [22.4–32.7]	119	36.6 [31.5–42.0]
	7^th^	256	45.6 [41.5–49.8]	108	41.4 [35.5–47.5]	148	49.3 [43.7–55.0]
	8^th^	284	54.3 [50.0–58.5]	141	59.0 [52.6–65.1]	143	50.4 [44.5–56.2]
Total		873	39.2 [37.2–41.3]	385	37.0 [34.1–40.0]	488	41.2 [38.4–44.0]
Total missing values		36	1.6 [1.2–2.2]	14	1.3 [0.8–2.2]	22	1.8 [1.2–2.8]
Drunkenness in the last 30 days	5^th^	9	1.7 [0.9–3.2]	5	2.0 [0.8–4.6]	4	1.4 [0.5–3.8]
	6^th^	6	1.0 [0.4–2.2]	2	0.7 [0.2–2.8]	4	1.2 [0.5–3.2]]
	7^th^	9	1.6 [0.8–3.1]	4	1.5 [0.6–4.0]	5	1.7 [0.7–3.9]
	8^th^	23	4.3 [2.9–6.4]	12	5.0 [2.9–8.6]	11	3.8 [2.1–6.7]
Total		47	2.1 [1.6–2.8]	23	2.2 [1.5–3.3]	24	2.0 [1.4–3.0]
Total missing values		27	1.2 [0.8–1.7]	12	1.1 [0.6–2.0]	15	1.2 [0.8–2.1]
Binge drinking in the last 30 days	5^th^	14	2.6 [1.6–4.4]	8	3.1 [1.6–6.1]	6	2.2 [1.0–4.8]
	6^th^	16	2.6 [1.6–4.2]	9	3.1 [1.6–5.9]	7	2.2 [1.0–4.5]
	7^th^	24	4.3 [2.9–6.3]	12	4.6 [2.6–7.9]	12	4.0 [2.3–6.9]
	8^th^	43	8.1 [6.1–10.8]	29	12.1 [8.5–16.9]	14	4.8 [2.9–8.0]
Total		97	4.3 [3.6–5.3]	58	5.5 [4.3–7.1]	39	3.3 [2.4–4.5]
Total missing values		26	1.2 [0.8–1.7]	9	0.9 [0.4–1.6]	17	1.4 [0.9–2.3]
Marijuana use in the last 30 days	5^th^	9	1.7 [0.9–3.2]	4	1.6 [0.6–4.1]	5	1.8 [0.8–4.3]
	6^th^	12	2.0 [1.1–3.4]	4	1.4 [0.5–3.7]	8	2.5 [1.2–4.9]
	7^th^	12	2.2 [1.2–3.8]	3	1.2 [0.4–3.5]	9	3.0 [1.6–5.7]
	8^th^	27	5.1 [3.5–7.4]	14	5.9 [3.5–9.7]	13	4.5 [2.6–7.6]
Total		60	2.7 [2.1–3.5]	25	2.4 [1.6–3.5]	35	3.0 [2.1–4.1]
Total missing values		33	1.5 [1.0–2.0]	11	1.0 [0.6–1.9]	22	1.8 [1.2–2.8]
Marijuana use in the last year	5^th^	10	1.9 [1.0–3.5]	5	2.0 [0.8–4.6]	5	1.9 [0.8–4.4]
	6^th^	21	3.4 [2.3–5.2]	7	2.4 [1.2–5.0]	14	4.3 [2.6–7.2]
	7^th^	27	4.8 [3.3–7.0]	10	3.9 [2.1–7.0]	17	5.7 [3.6–9.0]
	8^th^	41	7.8 [5.8–10.4]	21	8.8 [5.8–13.1]	20	6.9 [4.5–10.5]
Total		99	4.5 [3.7–5.4]	43	4.1 [3.1–5.5]	56	4.8 [3.7–6.1]
Total missing values		43	1.9 [1.4–2.6]	13	1.2 [0.7–2.1]	30	2.5 [1.7–3.5]
Lifetime marijuana use	5^th^	14	2.7 [1.6–4.5]	4	1.6 [0.6–4.1]	10	3.7 [2.0–6.8]
	6^th^	35	5.7 [4.2–7.9]	10	3.5 [1.9–6.4]	25	7.8 [5.3–11.2]
	7^th^	42	7.6 [5.6–10.1]	19	7.3 [4.7–11.2]	23	7.8 [5.2–11.4]
	8^th^	69	13.0 [10.4–16.2]	37	15.4 [11.4–20.6]	32	11.1 [7.9–15.3]
Total		160	7.2 [6.2–8.4]	70	6.7 [5.4–8.4]	90	7.7 [6.3–9.3]
Total missing values		46	2.0 [1.5–2.7]	14	1.3 [0.8–2.2]	32	2.7 [1.9–3.7]

#### Evidence of the internal structure of the EU-Dap questionnaire

All of the original EU-Dap subscales had acceptable KMO values to perform factor analysis. Regarding their internal reliability, all subscales had an acceptable coefficient (≥0.65), except for the decision-making skills subscale (ω = 0.59).

Applying the factor loading criteria mentioned in the Methodology, the majority of 16 subscales kept all the items originally included in the construct, except four subscales which ended up with a reduced number of items: self-esteem, nine items (1 item was removed), family functioning, 16 items (4 items were removed), assertiveness, five items (1 item was removed), and normative beliefs, three items (2 removed). See Table 3 and S2 Table.

**Table 3:** Original structure of subscales before removing some items.

	Number of Factors	Number of Items	KMO	Omega Reliability	Decision* (#item)**
Positive and negative beliefs about tobacco use	2	8	0.74	0.77	Keep all items; two subscales: a positive and a negative dimension.
Positive and negative beliefs about alcohol use	2	10	0.84	0.86	Keep all items; two subscales: a positive and a negative dimension.
Positive and negative beliefs about marijuana use	2	10	0.84	0.88	Keep all items; two subscales: a positive and a negative dimension.
Positive and Negative Attitudes towards Drugs	2	11	0.84	0.81	Keep all items; two subscales: a positive and a negative dimension.
Self-esteem	2	10	0.77	0.76	Item (#9) deleted; two subscales: a positive and a negative dimension.
Future substance use	1	6	0.85	0.95	Keep all items; one subscale.
Poor problem-solving skills	1	5	0.70	0.65	Keep all items; one subscale.
Substance abuse index	1	11	0.91	0.92	Keep all items; one subscale.
Parental Involvement	1	4	0.81	0.78	Keep all items; one subscale.
Family functioning	1	20	0.88	0.83	Items (#9, #13, #17, and #18,) deleted; one subscale.
School bonding	1	5	0.75	0.80	Keep all items; one subscale.
Risk Perception	1	8	0.82	0.86	Keep all items; one subscale.
Assertiveness	1	6	0.77	0.71	Item (#3) deleted; one subscale.
Normative beliefs	1	5	0.70	0.69	Items (#1, #2) deleted; one subscale.
Refusal skills	1	3	0.73	0.86	Keep all items; one subscale.
Decision-making skills	1	5	0.52	0.59	Keep all items; one subscale.

Note

* The number of subscales created is based on the number of factors, and the dimensions with two factors were based on 2-correlated models.

** The #item is related to the position of the item in the scale, and this position does not refer to the actual position in the questionnaire.

The subscales “positive and negative beliefs about tobacco use”, “positive and negative beliefs about alcohol use”, “positive and negative beliefs about marijuana use”, “positive and negative attitudes towards drugs” and “self-esteem” had two oppositive dimensions; therefore, two subscales for each construct were created. See Table 3.

Finally, the items that did not load into any of the subscales, we decided to keep them in the questionnaire and perform association analyses using them as individual items. We considered the content of these items valuable, and they were assessed as risk or protective factors for substance use outcomes.

#### Evidence of internal structure of the final scales and subscales of the EU-Dap questionnaire

CFI index showed acceptable or good adjustment for positive and negative beliefs about marijuana use, future substance use, substance abuse index, parental involvement, normative beliefs and refusal skills. Regarding the SRMR, good or acceptable fit was found for the following subscales: positive and negative beliefs about tobacco, self-esteem, poor problem-solving skills, parental involvement, school bonding, assertiveness, normative beliefs, and refusal skills. Additionally, the RMSEA index was good or acceptable for substance abuse index, parental involvement, family functioning, normative beliefs, and refusal skills. Finally, most subscales had either good or acceptable internal reliability (range 0.65 to 0.95), except for decision-making skills, with ω = 0.59. See Table 4. Further information about matrixes of the subscales and items can be found in S4 Table.

**Table 4:** Internal structure of the final scales and subscales of the EU-Dap questionnaire.

Scale	*Number of Factors	Number of items	Omega Reliability	CFI	SRMR	RMSEA
Positive and Negative Beliefs about Tobacco use	2	8	0.77	0.93	0.10	0.10
Positive subscale	1	4	0.74			
Negative subscale	1	4	0.83			
Positive and negative beliefs about alcohol use	2	10	0.86	0.94	0.11	0.09
Positive subscale	1	5	0.89			
Negative subscale	1	5	0.87			
Positive and negative beliefs about marijuana use	2	10	0.88	0.97	0.14	0.10
Positive subscale	1	4	0.89			
Negative subscale	1	6	0.92			
Positive and Negative Attitudes towards Drugs	2	11	0.81	0.91	0.11	0.09
Positive subscale	1	4	0.74			
Negative subscale	1	7	0.82			
Self-esteem	2	9	0.77	0.90	0.07	0.15
Positive subscale	1	5	0.80			
Negative subscale	1	4	0.70			
Future substance use	1	6	0.95	0.99	0.12	0.09
Poor problem-solving skills	1	5	0.65	0.90	0.07	0.15
Substance abuse index	1	11	0.92	0.97	0.13	0.05
Parental Involvement	1	4	0.78	0.97	0.04	0.06
Family functioning	1	16	0.65	0.82	0.14	0.08
School bonding	1	5	0.80	0.93	0.09	0.16
Risk Perception	1	8	0.86	0.88	0.14	0.10
Assertiveness	1	5	0.74	0.94	0.05	0.09
Normative beliefs	1	3	0.88	1.00	0.00	0.00
Refusal skills	1	3	0.86	1.00	0.00	0.00
Decision-making skills	1	5	0.59	0.45	0.13	0.28

Note

*The dimensions (positive subscale and negative subscale) were based on 2-correlated models. Comparative adjustment index (CFI) [≥ 0.97 = good adjustment; 0.95–0.96 = acceptable adjustment], the standardized mean square residual root (SRMR) [≤ 0.05 = good fit; 0.06–0.10 acceptable fit], the root mean square error of approximation (RMSEA) [≤ 0.05 = good fit; 0.06–0.08 = acceptable fit]; and N/A = Not applicable because the items did not converge in factor analysis.

#### Associations of EU-Dap risk and protective factors and substance use

The results of the univariable associations are presented in S5 Table. Most of these associations were significant and included in further analyses.

Regarding multivariable analyses, negative beliefs about alcohol use decreased the odds of 30-day prevalence alcohol and the 30-day prevalence of binge drinking in females. Positive attitudes towards drugs increased the odds of 30-day prevalence of marijuana use, and negative beliefs about marijuana decreased the risk of 12-month prevalence of marijuana use. The Future substance use subscale score increased the risk of alcohol use and binge drinking among females in the last 30 days. The substance abuse index increased the risk of marijuana use in the two time periods studied. On the other hand, school bonding decreased the odds of tobacco, alcohol, and drunkenness 30-day prevalence, and risk perception decreased the odds of 30-day prevalence of marijuana use. Higher scores in the normative beliefs subscale increased the risk for the three substances: 30-day alcohol, 30-day tobacco, and 12-month marijuana prevalence. Finally, refusal skills decreased the risk of alcohol and drunkenness 30-day prevalence. See Table 5.

**Table 5:** Multivariable associations of EU-Dap subscales and substance use.

EU-Dap subscales	Tobacco use in the last 30 days	Alcohol use in the last 30 days	Drunk in the last 30 days	Binge drinking in the last 30 days Females (1)	Binge drinking in the last 30 days Males (1)	Marijuana use in the last 30 days	Marijuana use in the last 12 months
	OR [95% CI]	OR [95% CI]	OR [95% CI]	OR [95% CI]	OR [95% CI]	OR [95% CI]	OR [95% CI]
	p-value	p-value	p-value	p-value	p-value	p-value	p-value
**Positive beliefs about tobacco use**	0.91 [0.74–1.13]						
	0.410						
**Negative beliefs about tobacco use**	0.85 [0.70–1.04]						
	0.107						
**Positive beliefs about alcohol use**		1.04 [0.96–1.13]	1.13 [0.92–1.38]	0.99 [0.82–1.19]	1.10 [0.96–1.27]		
		0.292	0.234	0.892	0.171		
**Negative beliefs about alcohol use**		0.88** [0.82–0.95]	0.86 [0.69–1.07]	0.78* [0.65–0.95]	0.87 [0.76–1.01]		
		0.001	0.185	0.013	0.063		
**Positive beliefs about marijuana use**						1.08 [0.88–1.34]	1.19 [0.98–1.44]
						0.455	0.081
**Negative beliefs about marijuana use**						0.90 [0.77–1.04]	0.81* [0.71–0.93]
						0.139	0.002
**Positive attitudes towards drugs**						1.53* [1.13–2.06]	1.13 [0.88–1.45]
						0.006	0.333
**Negative attitudes towards drugs**						0.88 [0.74–1.05]	0.99 [0.84–1.15]
						0.166	0.855
**Positive Self esteem**	1.01 [0.79–1.30]	1.07 [0.96–1.19]	1.33 [0.96–1.85]	0.96 [0.76–1.19]			
	0.917	0.210	0.089	0.688			
**Negative Self esteem**	1.06 [0.87–1.29]			1.16 [0.91–1.48]	1.08 [0.88–1.34]	0.86 [0.65–1.14]	1.03 [0.83–1.29]
	0.579			0.216	0.467	0.298	0.782
**Future substance use**	1.14 [1.01–1.28]	1.16** [1.08–1.24]	1.12 [0.94–1.34]	1.28* [1.09–1.50]	1.10 [0.98–1.24]	1.08 [0.92–1.28]	1.10 [0.95–1.28]
	0.041	0.000	0.189	0.002	0.113	0.352	0.190
**Poor problem-solving skills**	1.06 [0.88–1.29]	0.97 [0.87–1.07]		1.06 [0.84–1.35]	1.05 [0.87–1.27]	0.95 [0.71–1.27]	0.99 [0.78–1.27]
	0.529	0.501		0.618	0.625	0.707	0.955
**Substance abuse index**	0.99 [0.59–1.66]	0.86 [0.65–1.11]	0.98 [0.66–1.46]	1.44 [0.93–2.24]		2.63** [1.49–4.65]	2.56** [1.50–4.37]
	0.969	0.243	0.933	0.106		0.001	0.001
**Parental Involvement**	1.04 [0.78–1.39]	0.97 [0.86–1.11]	0.91 [0.65–1.28]	1.06 [0.81–1.38]		1.41 [0.98–2.05]	1.25 [0.92–1.68]
	0.782	0.684	0.596	0.686		0.065	0.149
**Family functioning**	0.98 [0.89–1.08]	1.01 [0.97–1.06]	1.01 [0.89–1.15]	1.08 [0.97–1.20]		0.95 [0.85–1.05]	0.94 [0.86–1.03]
	0.735	0.578	0.843	0.161		0.324	0.168
**School bonding**	0.77* [0.64–0.93]	0.90* [0.81–0.99]	0.79* [0.62–0.99]	0.94 [0.74–1.20]			0.84 [0.67–1.04]
	0.006	0.033	0.041	0.620			0.105
**Risk Perception**	1.13 [0.88–1.43]					0.74* [0.58–0.94]	0.82 [0.66–1.02]
	0.335					0.013	0.080
**Assertiveness**	0.97 [0.79–1.20]		1.24 [0.88–1.74]				
	0.804		0.214				
**Normative beliefs**	1.52** [1.23–1.87]	1.28** [1.12–1.47]	1.19 [0.85–1.66]	1.33* [1.01–1.76]	0.98 [0.81–1.20]	1.21 [0.91–1.61]	1.47** [1.17–1.85]
	0.000	0.000	0.306	0.045	0.875	0.182	0.001
**Refusal skills**	0.89 [0.70–1.12]	0.79** [0.70–0.90]	0.75 [0.55–1.02]	0.84 [0.65–1.09]	0.85 [0.69–1.05]	1.40 [0.94–2.09]	1.11 [0.85–1.47]
	0.326	0.000	0.070	0.196	0.139	0.097	0.427
**Decision-making skills**	0.91 [0.70–1.17]			1.08 [0.83–1.41]	0.91 [0.72–1.14]	0.83 [0.64–1.09]	1.00 [0.76–1.31]
	0.445			0.561	0.141	0.185	0.991

Note: All associations were adjusted by gender and age. Empty cells indicate that the variables did not enter into the model.

*p≤0.05 and

**p≤0.001. (1) For binge drinking, there are two different definitions according to gender. In females, the variable is defined using 4 or more drinks on the same occasion (2 hours); while in males, the variable is defined as 5 or more drinks on the same occasion (2 hours).

The effect size estimation for one of the relevant associations explored in this study, “Alcohol use in the last 30 days” as the dependent variable and “Normative Beliefs” as an independent variable, was Cohen’s d of -0.60 (95% CI = -0.41: -0.79), which is considered a medium effect size [47].

## Discussion

This is the first validation of the EU-Dap questionnaire in an early adolescent population in Spanish-speaking Latin American countries. The results of our study showed that the EU-Dap questionnaire has good psychometric properties. Substance use questions were well understood and seemed to adequately capture the consumption of different drugs. Regarding the subscales of risk and protective factors, the evidence of construct validity using confirmatory factor analysis showed that most of the final subscales had good or adequate goodness of fit adjustments. Regarding reliability, all of the final subscales had good or acceptable internal consistency according to the omega coefficient [48]. Association analyses showed different risk and protective factors. Still, above all, normative beliefs was the most consistent, and it was strongly associated with the three substances of interest: tobacco, alcohol, and marijuana. All these association results are important because several preventive interventions aim to increase some of these personal protective factors; therefore, this questionnaire may help to evaluate the effectiveness of these interventions and the potential mediating factors explaining the effect of the interventions.

The general content of the questionnaire was well understood, and only words and small changes in the structure of the questions were required. Similar results were found in the validation in Brazil [27]. These results did not diminish the importance of completing a systematic approach to culturally adapt a questionnaire using qualitative data as was our case and in Brazil. In Brazil, environmental characteristics that facilitated (e.g., good discipline) or hindered (e.g., crowded classrooms) the completion of the questionnaires were important issues, but these features were not reported as important in our focus groups. Most students had good behavior during the application of the instrument, but we have to consider these potential environment features that may affect its application, especially when the implementation progresses to the scaling-up stage.

Regarding the factor structure of the EU-Dap questionnaire, to our knowledge, no other publications are exploring the validity of the different subscales contained in the instrument. The fact that most subscales exploring risk and protective factors had good item structure is important, considering that some of these variables might be used to study the mediating effect of the Unplugged intervention. Having a valid instrument to assess this and other variables, will help to conduct similar analyses after we complete the cRCT in schools in Chile or other research teams conduct other evaluations of different programs. Similarly, it is crucial to have reliable instruments, and our results support this idea.

We also explored the associations between risk and protective factors and substance use outcomes. This approach allowed us to examine the association of potentially modifiable factors with substance use and provide support for using these scales to evaluate the effectiveness of preventive interventions. For example, normative beliefs had one of the strongest associations in our study. This variable was considered a mediator factor in the effectiveness of the Unplugged program in Europe, and several activities of the program aimed to improve this factor [43].

Comparing our results with another study among Chilean adolescents that measured the impact of risk and protective factors in the use of tobacco, alcohol, and marijuana [49], both highlighted the importance of normative beliefs as a risk factor. Having the perception that most friends use substances increased the risk of drug use among the students. This finding is supported by other studies [50, 51] and emphasizes that students may be more influenced by their beliefs or the interpretation of reality than by the actual prevalence of drug use among friends [52, 53]. Interventions aiming to increase critical thinking and fact-checking skills may help to reduce the impacts of these normative beliefs [19, 54].

Another critical variable was school bonding, which reduced the risk for tobacco and alcohol use in the last 30 days. This relationship has already been reported in Chile [55] and elsewhere [56, 57]. Interestingly, the #Tamojunto, the Brazilian version of the Unplugged program, reduced the bullying experience, especially among girls 11–13 years old at 9 months of follow-up [58]. Even though these results were not sustained at 21 months, these findings partially support the idea that school bonding may play a role in the effectiveness of these interventions.

Furthermore, building refusal skills may also appear to be a protective factor [25]. Including activities in preventive interventions where students practice these skills may help to deal with peer pressure, especially during social events, which are the main source of influence for the onset of substance use [43].

We also highlight the importance of negative beliefs about alcohol and marihuana as potential protectives factors, findings also found in a Brazilian Unplugged study [59]. Furthermore, according to a systematic review [60], other programs like “Towards No Drug Abuse” from the United States and “School-Based Education” from Germany that included “beliefs towards substances” in their objectives seem to have some positive effect on substance use.

This study has some limitations. First, this was a self-reported questionnaire, so information bias and social desirability bias may exist. Therefore, the substance use prevalence figures may have been overestimated or underestimated. Second, this is a cross-sectional study, so no causality may have been claimed regarding the associations [61]. Third, the cultural and linguistic adaptation in Study 1 was made only among students coming from middle-income families, which may have introduced some bias in the understanding of the questions. However, there was no report of difficulties of understanding the questions in the students coming from different socioeconomic backgrounds in Study 2. Fourth, since we had a higher proportion of boys participating in the study, a potential gender bias may have been introduced in the results. Among the strengths of our research, we had a large sample size (N = 2261), and a good representation of different socioeconomic levels. Furthermore, we adapted the questionnaire and gathered information to understand the questions, including the opinions of different aged students; we also used multiple sources of validity, such as confirmatory factor analyses; and we explored the associations between the resulting EU-Dap subscales and other risk and protective factor variables.

As previously mentioned, the EU-Dap questionnaire was developed to evaluate a preventive substance use program. According to these findings, we have a valid and reliable questionnaire available to evaluate the effectiveness of the “Yo Sé Lo Que Quiero” (Unplugged) in Chile and other Spanish speaking countries, or any other intervention aiming to reduce risk factors such as normative beliefs and increase protective factors such as negative beliefs about substance use, refusal skills, and school bonding.

## Supporting information

S1 Questionnaire Original EU-Dap questionnaire. (DOCX)

S2 Questionnaire Chilean version of EU-Dap questionnaire. (DOCX)

S1 Table Family structure. (DOCX)

S2 Table Description of scales. (DOCX)

S3 Table Other risk and protective factors. (DOCX)

S4 Table Subscales correlations. (DOCX)

S5 Table Univariable associations of EU-Dap risk and protective and substance use. (DOCX)

## References

[1] Degenhardt L, Stockings E, Patton G, Hall WD, Lynskey M. The increasing global health priority of substance use in young people. Lancet Psychiatry. 2016;3(3):251–64. Epub 2016/02/26. doi: 10.1016/S2215-0366(15)00508-8 . 26905480

[2] Hong SA, Peltzer K. Early Adolescent Patterns of Alcohol and Tobacco Use in Eight Association of South-East Asian Nations (ASEAN) Member States. Subst Use Misuse. 2019;54(2):288–96. Epub 2018/11/23. doi: 10.1080/10826084.2018.1517797 . 30463459

[3] Lebel C, Beaulieu C. Longitudinal development of human brain wiring continues from childhood into adulthood. J Neurosci. 2011;31(30):10937–47. Epub 2011/07/29. doi: 10.1523/JNEUROSCI.5302-10.2011 ; PubMed Central PMCID: PMC6623097. 21795544 PMC6623097

[4] Meruelo AD, Castro N, Cota CI, Tapert SF. Cannabis and alcohol use, and the developing brain. Behav Brain Res. 2017;325(Pt A):44–50. Epub 2017/02/23. doi: 10.1016/j.bbr.2017.02.025 ; PubMed Central PMCID: PMC5406224. 28223098 PMC5406224

[5] Squeglia LM, Gray KM. Alcohol and Drug Use and the Developing Brain. Curr Psychiatry Rep. 2016;18(5):46. Epub 2016/03/18. doi: 10.1007/s11920-016-0689-y ; PubMed Central PMCID: PMC4883014. 26984684 PMC4883014

[6] Wetherill R, Tapert SF. Adolescent brain development, substance use, and psychotherapeutic change. Psychol Addict Behav. 2013;27(2):393–402. Epub 2012/06/27. doi: 10.1037/a0029111 ; PubMed Central PMCID: PMC3700608. 22732057 PMC3700608

[7] Hynes M, Clarke P, Cumsille F, Araneda J, Ahumada G. Informe sobre el consumo de drogas en las Américas 2019 2019.

[8] Pizarro E, José M, Nicolás R. Décimo Segundo Estudio Nacional de drogas en población escolar de Chile, 2017 2019.

[9] Esmaeelzadeh S, Moraros J, Thorpe L, Bird Y. The association between depression, anxiety and substance use among Canadian post-secondary students. Neuropsychiatr Dis Treat. 2018;14:3241–51. Epub 2018/12/13. doi: 10.2147/NDT.S187419 ; PubMed Central PMCID: PMC6260190. 30538482 PMC6260190

[10] Hall WD, Patton G, Stockings E, Weier M, Lynskey M, Morley KI, et al. Why young people’s substance use matters for global health. Lancet Psychiatry. 2016;3(3):265–79. Epub 2016/02/26. doi: 10.1016/S2215-0366(16)00013-4 . 26905482

[11] Davidson LL, Grigorenko EL, Boivin MJ, Rapa E, Stein A. A focus on adolescence to reduce neurological, mental health and substance-use disability. Nature. 2015;527(7578):S161–6. Epub 2015/11/19. doi: 10.1038/nature16030 . 26580322

[12] Superintendencia de Salud. Casos GES (AUGE) acumulados a diciembre de 2019 2020 [cited 2020 17 Jun]. Available from: http://www.supersalud.gob.cl/documentacion/666/w3-article-19244.html.

[13] Fondo Nacional de Salud. Aranceles Modalidad Atención Institucional 2020 [cited 2020 17 Jun]. Available from: https://www.fonasa.cl/sites/fonasa/prestadores/modalidad-atencion-institucional.

[14] Egresos Hospitalarios [Internet]. 2017 [cited 19 Dic]. Available from: http://www.deis.cl/estadisticas-egresoshospitalarios/.

[15] Lize SE, Iachini AL, Tang W, Tucker J, Seay KD, Clone S, et al. A Meta-analysis of the Effectiveness of Interactive Middle School Cannabis Prevention Programs. Prev Sci. 2017;18(1):50–60. Epub 2016/10/28. doi: 10.1007/s11121-016-0723-7 ; PubMed Central PMCID: PMC5680036. 27785662 PMC5680036

[16] Agabio R, Trincas G, Floris F, Mura G, Sancassiani F, Angermeyer MC. A Systematic Review of School-Based Alcohol and other Drug Prevention Programs. Clin Pract Epidemiol Ment Health. 2015;11(Suppl 1 M6):102–12. Epub 2015/04/04. doi: 10.2174/1745017901511010102 ; PubMed Central PMCID: PMC4378029. 25834630 PMC4378029

[17] Faggiano F, Galanti MR, Bohrn K, Burkhart G, Vigna-Taglianti F, Cuomo L, et al. The effectiveness of a school-based substance abuse prevention program: EU-Dap cluster randomised controlled trial. Prev Med. 2008;47(5):537–43. Epub 2008/07/29. doi: 10.1016/j.ypmed.2008.06.018 . 18657569

[18] Faggiano F, Vigna-Taglianti F, Burkhart G, Bohrn K, Cuomo L, Gregori D, et al. The effectiveness of a school-based substance abuse prevention program: 18-month follow-up of the EU-Dap cluster randomized controlled trial. Drug Alcohol Depend. 2010;108(1–2):56–64. Epub 2010/01/19. doi: 10.1016/j.drugalcdep.2009.11.018 . 20080363

[19] Vigna-Taglianti FD, Galanti MR, Burkhart G, Caria MP, Vadrucci S, Faggiano F. “Unplugged,” a European school-based program for substance use prevention among adolescents: overview of results from the EU-Dap trial. New Dir Youth Dev. 2014;2014(141):67–82, 11–2. Epub 2014/04/23. doi: 10.1002/yd.20087 . 24753279

[20] Faggiano F, Minozzi S, Versino E, Buscemi D. Universal school-based prevention for illicit drug use. Cochrane Database Syst Rev. 2014;2014(12):Cd003020. Epub 2014/12/02. doi: 10.1002/14651858.CD003020.pub3 ; PubMed Central PMCID: PMC6483627 evaluation of and data extraction for the related papers. SM EV,DB have no conflicts of interest. 25435250 PMC6483627

[21] Knight JR, Shrier LA, Bravender TD, Farrell M, Vander Bilt J, Shaffer HJ. A new brief screen for adolescent substance abuse. Arch Pediatr Adolesc Med. 1999;153(6):591–6. Epub 1999/06/05. doi: 10.1001/archpedi.153.6.591 . 10357299

[22] Saunders JB, Aasland OG, Babor TF, de la Fuente JR, Grant M. Development of the Alcohol Use Disorders Identification Test (AUDIT): WHO Collaborative Project on Early Detection of Persons with Harmful Alcohol Consumption—II. Addiction. 1993;88(6):791–804. Epub 1993/06/01. doi: 10.1111/j.1360-0443.1993.tb02093.x 8329970

[23] Bashford J, Flett R, Copeland J. The Cannabis Use Problems Identification Test (CUPIT): development, reliability, concurrent and predictive validity among adolescents and adults. Addiction. 2010;105(4):615–25. Epub 2010/04/21. doi: 10.1111/j.1360-0443.2009.02859.x . 20403014

[24] Santis R, Garmendia ML, Acuña G, Alvarado ME, Arteaga O. The Alcohol Use Disorders Identification Test (AUDIT) as a screening instrument for adolescents. Drug Alcohol Depend. 2009;103(3):155–8. Epub 2009/05/09. doi: 10.1016/j.drugalcdep.2009.01.017 . 19423240

[25] Giannotta F, Vigna-Taglianti F, Rosaria Galanti M, Scatigna M, Faggiano F. Short-term mediating factors of a school-based intervention to prevent youth substance use in Europe. J Adolesc Health. 2014;54(5):565–73. Epub 2013/12/18. doi: 10.1016/j.jadohealth.2013.10.009 . 24332392

[26] Faggiano F, Richardson C, Bohrn K, Galanti MR. A cluster randomized controlled trial of school-based prevention of tobacco, alcohol and drug use: the EU-Dap design and study population. Prev Med. 2007;44(2):170–3. Epub 2006/11/07. doi: 10.1016/j.ypmed.2006.09.010 . 17084887

[27] Prado MC, Schneider DR, Sañudo A, Pereira AP, Horr JF, Sanchez ZM. Transcultural Adaptation of Questionnaire to Evaluate Drug Use Among Students: The Use of the EU-Dap European Questionnaire in Brazil. Subst Use Misuse. 2016;51(4):449–58. Epub 2016/02/20. doi: 10.3109/10826084.2015.1117108 . 26894657

[28] Sanchez ZM, Valente JY, Sanudo A, Pereira APD, Cruz JI, Schneider D, et al. The #Tamojunto Drug Prevention Program in Brazilian Schools: a Randomized Controlled Trial. Prev Sci. 2017;18(7):772–82. Epub 2017/04/01. doi: 10.1007/s11121-017-0770-8 . 28361199

[29] Carvajal A, Centeno C, Watson R, Martínez M, Rubiales AS. [How is an instrument for measuring health to be validated?]. An Sist Sanit Navar. 2011;34(1):63–72. Epub 2011/05/03. doi: 10.4321/s1137-66272011000100007 . 21532647

[30] Bolarinwa OA. Principles and methods of validity and reliability testing of questionnaires used in social and health science researches. Niger Postgrad Med J. 2015;22(4):195–201. Epub 2016/01/19. doi: 10.4103/1117-1936.173959 . 26776330

[31] Strauss A, Corbin J. Bases de la investigación cualitativa: técnicas y procedimientos para desarrollar la teoría fundamentada: Universidad de Antioquia; 2016.

[32] Harrington D. Confirmatory factor analysis: Oxford university press; 2009.

[33] De Vaus D, de Vaus D. Surveys in social research: Routledge; 2013.

[34] Inchley J, Currie D. Growing up unequal: gender and socioeconomic differences in young people’s health and well-being. Health Behaviour in School-aged Children (HBSC) study: international report from the. 2013;2014:2–3.

[35] Ministerio de Educación. Estadísticas de la Educación 2019 [cited 2021 6 Jun]. Available from: https://centroestudios.mineduc.cl/wp-content/uploads/sites/100/2019/11/ANUARIO-2018-PDF-WEB-FINALr.pdf.

[36] Villalobos Dintrans C, Wyman San Martín I, Schiele Muñoz B, Godoy Ossa F. Composición de género en establecimientos escolares chilenos:¿ Afecta el rendimiento académico y el ambiente escolar? Estudios pedagógicos (Valdivia). 2016;42(2):379–94.

[37] Agencia de Calidad de la Educación. Metodología de construcción de grupos socioeconómicos pruebas SIMCE 2013 2013 [cited 2020 18 Jun]. Available from: http://archivos.agenciaeducacion.cl/Metodologia_de_Construccion_de_Grupos_Socioeconomicos_Simce_2013.pdf.

[38] Lloret-Segura S, Ferreres-Traver A, Hernandez-Baeza A, Tomas-Marco I. Exploratory item factor analysis: A practical guide revised and updated. Anales de Psicología. 2014;30(3):1151–69.

[39] Cerny BA, Kaiser HF. A Study Of A Measure Of Sampling Adequacy For Factor-Analytic Correlation Matrices. Multivariate Behav Res. 1977;12(1):43–7. Epub 1977/01/01. doi: 10.1207/s15327906mbr1201_3 . 26804143

[40] Holgado–Tello FP, Chacón–Moscoso S, Barbero–García I, Vila–Abad E. Polychoric versus Pearson correlations in exploratory and confirmatory factor analysis of ordinal variables. Quality & Quantity. 2010;44(1):153.

[41] Horn JL. A RATIONALE AND TEST FOR THE NUMBER OF FACTORS IN FACTOR ANALYSIS. Psychometrika. 1965;30:179–85. Epub 1965/06/01. doi: 10.1007/BF02289447 . 14306381

[42] Hendrickson AE, White PO. Promax: A quick method for rotation to oblique simple structure. British journal of statistical psychology. 1964;17(1):65–70.

[43] McDonald R. Test Theory New York: Psychology Press; 2013 [cited 2020 June 18th].

[44] Schermelleh-Engel K, Moosbrugger H, Müller H. Evaluating the fit of structural equation models: Tests of significance and descriptive goodness-of-fit measures. Methods of psychological research online. 2003;8(2):23–74.

[45] Vadrucci S, Vigna-Taglianti FD, van der Kreeft P, Vassara M, Scatigna M, Faggiano F, et al. The theoretical model of the school-based prevention programme Unplugged. Glob Health Promot. 2016;23(4):49–58. Epub 2015/06/13. doi: 10.1177/1757975915579800 . 26062869

[46] Clark Goings T, Salas-Wright CP, Belgrave FZ, Nelson EJ, Harezlak J, Vaughn MG. Trends in binge drinking and alcohol abstention among adolescents in the US, 2002–2016. Drug Alcohol Depend. 2019;200:115–23. Epub 2019/05/24. doi: 10.1016/j.drugalcdep.2019.02.034 . 31121494

[47] Cohen J. Statistical power analysis for the behavioral sciences: Academic press; 2013.

[48] McNeish D. Thanks coefficient alpha, we’ll take it from here. Psychol Methods. 2018;23(3):412–33. Epub 2017/05/31. doi: 10.1037/met0000144 . 28557467

[49] Gaete J, Araya R. Individual and contextual factors associated with tobacco, alcohol, and cannabis use among Chilean adolescents: A multilevel study. J Adolesc. 2017;56:166–78. Epub 2017/03/05. doi: 10.1016/j.adolescence.2017.02.011 . 28259098

[50] Ra JS, Cho YH. Role of social normative beliefs as a moderating factor in smoking intention among adolescent girls in Korea. Nurs Health Sci. 2018;20(4):530–6. Epub 2018/09/14. doi: 10.1111/nhs.12565 . 30209874

[51] Leung RK, Toumbourou JW, Hemphill SA. The effect of peer influence and selection processes on adolescent alcohol use: a systematic review of longitudinal studies. Health Psychol Rev. 2014;8(4):426–57. Epub 2014/09/12. doi: 10.1080/17437199.2011.587961 . 25211209

[52] Wambeam RA, Canen EL, Linkenbach J, Otto J. Youth misperceptions of peer substance use norms: a hidden risk factor in state and community prevention. Prev Sci. 2014;15(1):75–84. Epub 2013/03/21. doi: 10.1007/s11121-013-0384-8 . 23512125

[53] Norton EC, Lindrooth RC, Ennett ST. How measures of perception from survey data lead to inconsistent regression results: evidence from adolescent and peer substance use. Health Econ. 2003;12(2):139–48. Epub 2003/02/04. doi: 10.1002/hec.705 . 12563661

[54] Mohammadi M, Ghaleiha A, Rahnama R. Effectiveness of a Peer-Led Behavioral Intervention Program on Tobacco Use-Related Knowledge, Attitude, Normative Beliefs, and Intention to Smoke among Adolescents at Iranian Public High Schools. Int J Prev Med. 2019;10:111. Epub 2019/07/31. doi: 10.4103/ijpvm.IJPVM_493_17 ; PubMed Central PMCID: PMC6592138. 31360358 PMC6592138

[55] Gaete J, Rojas G, Fritsch R, Araya R. Association between School Membership and Substance Use among Adolescents. Front Psychiatry. 2018;9:25–. doi: 10.3389/fpsyt.2018.00025 . 29479322 PMC5812301

[56] Weatherson KA, O’Neill M, Lau EY, Qian W, Leatherdale ST, Faulkner GEJ. The Protective Effects of School Connectedness on Substance Use and Physical Activity. Journal of Adolescent Health. 2018;63(6):724–31. doi: 10.1016/j.jadohealth.2018.07.002 30269908

[57] Mulla MM, Bogen KW, Orchowski LM. The mediating role of school connectedness in the associations between dating and sexual violence victimization and substance use among high school students. Preventive Medicine. 2020;139:106197. doi: 10.1016/j.ypmed.2020.106197 32652131

[58] Gusmões JDSP, Sañudo A, Valente JY, Sanchez ZM. Violence in Brazilian schools: Analysis of the effect of the #Tamojunto prevention program for bullying and physical violence. Journal of Adolescence. 2018;63:107–17. doi: 10.1016/j.adolescence.2017.12.003 29288995

[59] Sanchez ZM, Valente JY, Fidalgo TM, Leal AP, Medeiros PFP, Cogo-Moreira H. The role of normative beliefs in the mediation of a school-based drug prevention program: A secondary analysis of the #Tamojunto cluster-randomized trial. PLoS One. 2019;14(1):e0208072. Epub 2019/01/08. doi: 10.1371/journal.pone.0208072 ; PubMed Central PMCID: PMC6322758. 30615625 PMC6322758

[60] Lee NK, Cameron J, Battams S, Roche A. What works in school-based alcohol education: A systematic review. Health Education Journal. 2016;75(7):780–98.

[61] Cole DA, Maxwell SE. Testing mediational models with longitudinal data: questions and tips in the use of structural equation modeling. J Abnorm Psychol. 2003;112(4):558–77. Epub 2003/12/17. doi: 10.1037/0021-843X.112.4.558 . 14674869

