# Supplementary material for: Validation of the European Drug Addiction Prevention Trial Questionnaire (EU-Dap) for substance use screening and to assess risk and protective factors among early adolescents in Chile
Source: PLoS One. 2021 Oct 11;16(10):e0258288. doi: 10.1371/journal.pone.0258288 (PMC8504767; doi:10.1371/journal.pone.0258288)
Supplement: S1 Questionnaire — (DOCX) [file pone.0258288.s001.docx]

1. **You are** 1□ a boy _2_□ a girl

# In which year were you born? Year 19

1. **Which of the following people live in the same household with you?** Mark all that applies. 1□ Father

1□ Stepfather

1□ Mother

1□ Stepmother

1□ Brother(s) and/or sister(s) / step-brother(s) and/or sister(s)

1□ Grandparent(s) 1□ Other relative(s) 1□ Non-relative(s)

1. **Do you have siblings, including stepbrothers and stepsisters?** Mark all that applies. 1□ No

2□ Yes, older 3□ Yes, younger 4□ Yes, twins

# How many times (if any) have you smoked cigarettes?

**Mark one box for each line**

Number of times

0 1-2 3-5 6-9 10-19 20-29 30 or more

a) In your lifetime ................................... □ □ □ □ □ □ □

b) During the last 12 months .................. □ □ □ □ □ □ □

c) During the last 30 days ...................... □ □ □ □ □ □ □

1 2 3 4 5 6 7

# How many cigarettes do you usually smoke in a week?

If you smoke less than weekly or if you don’t smoke, please mark 0

Number of cigarettes a week

| 0 | 1-2 | 3-5 | 6-9 | 10-19 20 | or more |
| --- | --- | --- | --- | --- | --- |
| □ | □ | □ | □ | □ | □ |
| 1 | 2 | 3 | 4 | 5 | 6 |

1. **How likely is that each of the following would happen to you if you smoke cigarettes in the next month? Mark the answer that is closest to your opinion.**

**Very Likely Likely Unlikely Very Unlikely**

1. Get into trouble with parents................□ □ □ □
2. Have problems with my friends ...........□ □ □ □

c) Become an addict ...............................□ □ □ □

d) Have money problems ........................□ □ □ □

e) Feel more relaxed ...............................□ □ □ □

f) Have more fun ....................................□ □ □ □

g) Be more popular..................................□ □ □ □

h) Be more confident and outgoing..........□ □ □ □

1 2 3 4

# At present, how often do you drink anything alcoholic, such as beer, wine or spirits?

Try to include even those times when you only drink a small amount.

| Every Day | Every week | Every month | Seldom Never |
| --- | --- | --- | --- |
| □ | □ | □ | □ □ |
| 1 | 2 | 3 | 4 5 |

# How many times (if any) have you been drunk from drinking alcoholic beverages?

Mark one box for each line. Number of times

0 1-2 3-5 6-9 10-19 20-29 30 or more

a) In your lifetime ................................... □ □ □ □ □ □ □

b) During the last 12 months .................. □ □ □ □ □ □ □

c) During the last 30 days ...................... □ □ □ □ □ □ □

1 2 3 4 5 6 7

1. **How likely is that each of the following would happen to you if you drink alcohol in the next month? Mark the answer that is closest to your opinion.**

Very Likely Likely Unlikely Very Unlikely

a) Do badly in school...............................□ □ □ □

1. Get into trouble with parents................□ □ □ □
2. Have problems with my friends ...........□ □ □ □

d) Become an addict ...............................□ □ □ □

e) Have money problems ........................□ □ □ □

f) Feel more relaxed ...............................□ □ □ □

g) Have more fun ....................................□ □ □ □

h) Be more popular..................................□ □ □ □

i) Forget my troubles ..............................□ □ □ □

j) Be more confident and outgoing..........□ □ □ □

1 2 3 4

1. **How many times (if any) have you sniffed a substance (glue, petrol, paint thinner etc) to get high? Mark one box for each line. Number of times**

0 1-2 3-5 6-9 10-19 20-29 30 or

more

a) In your lifetime ................................... □ □ □ □ □ □ □

b) During the last 12 months .................. □ □ □ □ □ □ □

c) During the last 30 days ...................... □ □ □ □ □ □ □

1 2 3 4 5 6 7

# Have you ever heard of any of the following substances? _Mark_ _one_ _box_ _for_ _each_ _line._

| Yes | No |
| --- | --- |
| a) Tranquillisers or sedatives □ | □ |
| b) Marijuana or hashish □ | □ |
| c) LSD □ | □ |
| d) Amphetamines □ | □ |
| e) Crack □ | □ |
| f) Cocaine □ | □ |
| g) Relevin □ | □ |
| h) Heroin □ | □ |

|  | i) Ecstasy □ | □ |
| --- | --- | --- |
|  | j) GHB □ | □ |
|  | k) Methadone □ | □ |
|  | l) ”Magic mushrooms” □ | □ |
|  | m) Ketamine □ | □ |
|  | **1** | **2** |

1. **How many times (if any) have you used marijuana or hashish……? (never=0)**

Mark one box for each line. Number of times

0 1-2 3-5 6-9 10-19 20-29 30 or

more

a) In your lifetime ................................... □ □ □ □ □ □ □

b) During the last 12 months .................. □ □ □ □ □ □ □

c) During the last 30 days ...................... □ □ □ □ □ □ □

1 2 3 4 5 6 7

# Have you ever used any of the following drugs?

Mark one or more boxes for each line.

Yes, during Yes, during the Yes, during No the last 30 dayslast 12 months lifetime

| a) Tranquillisers/sedatives (without a doctor’s prescription)□ □ □ □ | | | |
| --- | --- | --- | --- |
| b) LSD or some other hallucinogens □ | □ | □ | □ |
| c) Amphetamines □ | □ | □ | □ |
| d) Crack □ | □ | □ | □ |
| e) Cocaine □ | □ | □ | □ |
| f) Relevin □ | □ | □ | □ |
| g) Heroin □ | □ | □ | □ |
| h) Ecstasy □ | □ | □ | □ |
| i) GHB □ | □ | □ | □ |
| j) Methadone □ | □ | □ | □ |
| k) ”Magic mushrooms” □ | □ | □ | □ |
| l) Ketamine □ | □ | □ | □ |
| 1 | 1 | 1 | 1 |

1. **How likely is that each of the following would happen to you if you take marijuana or other illegal substances in the next month? Mark the answer that is closest to your opinion.**

Very Likely Likely Unlikely Very Unlikely

a) Get into trouble with police ..................□ □ □ □

b) Have problems in school .....................□ □ □ □

1. Get into trouble with parents................□ □ □ □
2. Have problems with my friends ...........□ □ □ □

e) Become an addict ...............................□ □ □ □

f) Have money problems ........................□ □ □ □

g) Feel more relaxed ...............................□ □ □ □

h) Have more fun ....................................□ □ □ □

i) Be more popular..................................□ □ □ □

j) Be more confident and outgoing..........□ □ □ □

1 2 3 4

# How likely is it that you will be doing each of the following A YEAR FROM NOW?

Mark one box for each line.

Not Very

Very Likel Likely Unlikely Unlikely

a) smoke cigarettes................................... □ □ □ □

b) drink alcoholic beverages (beer, wine, spirits) □ □ □ □

c) get drunk............................................... □ □ □ □

1. smoke marijuana or hashish (pot, grass) □ □ □ □
2. sniff a substance (glue etc) to get high .. □ □ □ □

f) take illegal substances .......................... □ □ □ □

1 2 ... 3 4

# Here are some statements that people have made about illegal substances. How much do you agree with the following opinions on drugs?

Mark the answer that is closest to your opinion. Strongly Agree Disagree Strongly Agree Disagree

1. Using drugs can be a pleasant activity □ □ □ □
2. A young person should never try drugs □ □ □ □
3. Using drugs is fun □ □ □ □
4. Many things are much more risky than trying drugs □ □ □ □
5. Everyone who tries drugs eventually regrets it □ □ □ □
6. The laws about drugs should be made stronger □ □ □ □
7. Drug use is one of the biggest evils in the country □ □ □ □
8. Drugs help people to have experience life in full □ □ □ □
9. Schools should teach about the real hazards of

taking drugs □ □ □ □

1. The police should not be annoying young people

who are trying drugs □ □ □ □

1. To experiment with drugs is to give away control

of your life □ □ □ □

1 2 3 4

# For each statement below, please mark whether you think it is correct or not by checking the appropriate box.

Yes No Don’t know

1. Nicotine is the substance in cigarettes that causes lung cancer □ □ □
2. One needs to smoke several cigarettes per day

during many years to become addicted □ □ □

1. Women have lower tolerance to alcohol than men □ □ □
2. It takes about half an hour to eliminate from the body

the amount of alcohol contained in a can of strong beer □ □ □

1. Smoking marijuana does not cause physical dependence □ □ □
2. High consumption of hash or marijuana decreases

the production of sexual hormones □ □ □

1 2 3

# Here are some statements about your knowledge about some substances. How much do you agree with the following?

Mark the answer that is closest to your opinion. Strongly Strongly agree Agree Disagree disagree

I Know all I need to know about nicotine and its effects □

□

□

□

a)

1. I Know all I need to know about alcohol and its effects............... □ □ □ □
2. I Know all I need to know about other drugs and their effects..... □ □ □ □

1 2 3 4

1. **How much do you think PEOPLE RISK harming themselves (physically or in other ways), if they….. Mark one box for each line.**

No risk Slight risk Great risk Don’t

know

1. smoke cigarettes occasionally .............. □ □ □ □
2. smoke one or more packs of cigarettes per day □ □ □ □
3. have one or two drinks nearly each week □ □ □ □

d) drink alcohol every day......................... □ □ □ □

f) try inhalants (glue etc) once or twice .... □ □ □ □

1. try marijuana or hashish (cannabis, pot,

grass) once or twice ............................. □ □ □ □

1. smoke marijuana or hashish regularly .. □ □ □ □
2. use other drugs occasionally ................ □ □ □ □

1 2 3 4

# Do any of the following people smoke cigarettes? _Mark_ _one_ _box_ _for_ _each_ _line._

Smokes Smokes Does not Don’t Don’t have daily sometimes smoke know or see

this person

1. Mother □ □ □ □ □
2. Father □ □ □ □ □
3. Best friend □ □ □ □ □
4. Siblings □ □ □ □ □

1 2 3 4 5

1. **When you answer this question, think about the friends with whom you spend most of your leisure time. Mark one box for each line.**

None Less than About half More than All of Don’t half of of them half of them know them them

1. How many of them like school? □ □ □ □ □ □
2. How many of them do well at school? □ □ □ □ □ □
3. How many of them smoke cigarettes? □ □ □ □ □ □
4. How many of them get drunk? □ □ □ □ □ □
5. How many of them use marijuana or

Other drugs? □ □ □ □ □ □

1 2 3 4 5 6

# Does any of your siblings ……? _Mark_ _one_ _box_ _for_ _each_ _line._

Don’t have

Don’t any Yes No know siblings

a) drink alcoholic beverages (beer, wine, spirits) ............................ □ □ □ □

b) get drunk .................................................................................... □ □ □ □

c) smoke marijuana or hashish (pot, grass) . .................................. □ □ □ □

d) sniff substances (glue, petrol, paint thinner…) . .......................... □ □ □ □

e) take other drugs ......................................................................... □ □ □ □

1 2 3 4

# Do the following descriptions fit people around you? _Mark_ _the_ _answer_ _that_ _is_ _closest_ _to_ _your_ _opinion._

Strongly Agree Disagree Strongly Agree Disagree

1. My parents set clear rules □ □ □ □
2. My parents know where I am in the evenings □ □ □ □
3. I can easily get support from my father and/or mother □ □ □ □
4. It is very important for me not to disappoint my parents □ □ □ □
5. I can really get support from my best friend □ □ □ □

1 2 3 4

1. **In the following questions, you are to say whether you agree or disagree with each statement about your family. Mark the answer that is closest to your opinion.**

Strongly Disagree

Disagree Agree Strongly Agree

another

| a) In my family we really help and support one □ | | □ | □ | □ |
| --- | --- | --- | --- | --- |
| b) My family does not discuss its problems □ | | □ | □ | □ |
| c) We don’t often fight in my family | □ | □ | □ | □ |
| d) Each person’s duties are clearly set out in my family | □ | □ | □ | □ |
| e) In my family you can get away with almost anything | □ | □ | □ | □ |
| f) In my family we are full of life and good spirits | □ | □ | □ | □ |
| g) In my family its important for everyone to express their own opinion | □ | □ | □ | □ |
| h) In my family we hardly ever lose our tempers | □ | □ | □ | □ |
| i) There is strict punishment for anyone breaking the rules in my family | □ | □ | □ | □ |
| j) We can do whatever we want in my family | □ | □ | □ | □ |
| k) My family always does things together | □ | □ | □ | □ |
| l) There are a lot of discussions in my family | □ | □ | □ | □ |
| m) In my family we never hit each other | □ | □ | □ | □ |
| n) “Work before play” is the rule in my family | □ | □ | □ | □ |
| o) In my family we aren’t punished or told off when we do something wrong | □ | □ | □ | □ |
| p) We really get along well with each other | □ | □ | □ | □ |
| q) We don’t tell each other about our personal problems | □ | □ | □ | □ |
| r) In my family we don’t often criticize each other | □ | □ | □ | □ |
| s) Family members have strict ideas about what is right and what is wrong | □ | □ | □ | □ |
| t) We come and go as we want to in my family | □  1 | □  2 | □  3 | □  4 |

# If you wanted to drink alcohol (or already do), do you think your father and mother would allow you to do so? Mark one box for each line.

| Would allow (allows me) to drink  alcohol | Would not (does not) allow drinking  at home | Would not (does not) allow drinking  at all | Don’t know |
| --- | --- | --- | --- |
| □ | □ | □ | □ |
| 1 | 2 | 3 | 4 |

1. **If you wanted to smoke (or already do), do you think your father and mother would allow you to do so?** Mark one box for each line.

| Would allow (allows me) to smoke | Would not (does not) allow smoking  at home | Would not (does not) allow smoking  at all | Don’t know |
| --- | --- | --- | --- |
| □ | □ | □ | □ |
| 1 | 2 | 3 | 4 |

# How did your grades compare with those of your classmates during the last school year?

1□ Much better _2_□ Better _3_□ The same as most of them _4_□ Worse

# In your opinion, will you have improved your grades at the end of this school year?

1□ Yes _2_□ Probably yes _3_□ Probably no _4_□ No

# How do you feel about school at present?

1□ I like it a lot

2□ I like it a bit

3□ I don’t like it very much

4□ I don’t like it at all

# How much do you agree with the following descriptions of your school?

Mark one box for each line.

Strongly Agree Disagree Strongly agree disagree

1. The students in my class enjoy being together □ □ □ □
2. Most of the students in my class are kind and helpful □ □ □ □
3. Other students accept me as I am □ □ □ □
4. How I do in school matters a lot to me □ □ □ □
5. I have great respect for what my teachers tell me □ □ □ □

1 2 3 4

# Have you ever had any of the following problems in the last 12 months?

Mark all that applies for each line.

Yes for reasons Yes, because Yes, other than

Never of my because of alcohol or alcohol use my drug use drug use

a) Quarrel or argument ......................................................□ □ □ □

b) Scuffle or fight ...............................................................□ □ □ □

c) Accident or injury...........................................................□ □ □ □

1. Loss of money or other valuable items ..........................□ □ □ □
2. Damage to objects or clothing you owned .....................□ □ □ □
3. Problems in your relationship with (your) parents ..........□ □ □ □
4. Problems in your relationship with (your) friends ...........□ □ □ □
5. Problems in your relationship with (your) teachers ........□ □ □ □ i) Performed poorly at school............................................□ □ □ □ j) Victimized by robbery or theft ........................................□ □ □ □

k) Hospitalised or admitted to an emergency room............□ □ □ □

1 1 1 1

1. **There are several possible way to take decisions. How well do the following apply to you? Mark the answer that is closest to your opinion.**
2. When I have decided to do something, I always carry

Strongly Agree Disagree Strongly Agree Disagree

it through □ □ □ □

1. I often make up my mind without thinking of

the consequences □ □ □ □

1. I weigh up all the choices before I decide on

something □ □ □ □

1. I often regret something that I had decided □ □ □ □
2. When I decide on something it doesn't matter

what my friends think □ □ □ □

1 2 3 4

# Imagine yourself in each of the following situations. Some of them may be very familiar to you, some others less, so that you may feel less secure in answering. It is enough you do your best. Mark the answer that is closest to your opinion.

**Very likely likely unlikely very unlikely**

1. You and your best friend are at a party where you meet new people, and you feel you really want to get to know them. Someone offers you to smoke hash together. Your friend accepts. Do you?
2. You and the same friend are studying hard for an important test at school the day after. Both of you feel stressed and need to calm down. Your friend suggests a cigarette would help, and offers one. Do you accept?
3. The day after, you both pass the test, and feel now it is time to celebrate. Have still some pocket-money left, and the liquor store is nearby. Would you buy some alcohol (beer, wine) to celebrate?

□

□

□

□

□ □ □ □

| □ | □ | □ | □ |
| --- | --- | --- | --- |
| 1 | 2 | 3 | 4 |

# How much do you agree with the following descriptions of yourself?

**Mark the answer that is closest to your opinion.**

Strongly Agree Disagree Strongly

Agree Disagree

1. I feel that I have a number of good qualities □ □ □ □
2. I am able to do things as well as most other people □ □ □ □
3. At times I think I am no good at all □ □ □ □
4. Most boys and girls of my age are smarter than I am □ □ □ □
5. I am quite good at sports □ □ □ □
6. I feel very embarrassed when I have to say

something in class □ □ □ □

1. My being happy is important to my parents □ □ □ □
2. I worry a lot about silly things □ □ □ □
3. I often feel nervous over nothing at al □ □ □ □
4. I have plenty of interests and hobbies □ □ □ □

1 2 3 4

# Here are some statements about dealing with other people. _Mark_ _the_ _answer_ _that_ _is_ _closest_ _to_ _your_ opinion.

1. When someone tries to make you feel small, you should do

Strongly Agree Disagree Strongly Agree Disagree

the same to them □ □ □ □

1. There is point in letting people know you're angry with them □ □ □ □
2. The only way to deal with bullies is to let them know who

is in charge □ □ □ □

1. There are always ways of dealing with problems without

having to fight □ □ □ □

1. It is much better to 'fly off the handle' than to explain

things calmly □ □ □ □

1 2 3 4

1. **Imagine you would like to do the following things. How easy or difficult would you find it? Mark one box for each line.**

**I would like to…** very easy difficult very Easy difficult

1. Say something nice to a friend. □ □ □ □
2. Ask for a favour. □ □ □ □
3. Show someone that I like him/her. □ □ □ □
4. Say “no” when someone asks me to do something

I do not want to. □ □ □ □

1. Call for help when I have got problems. □ □ □ □
2. Help someone who needs help. □ □ □ □

1 2 3 4

Reprinted with permission from Federica Vigna-Taglianti, originally published in [2003].
