# Supplementary material for: Validation of the European Drug Addiction Prevention Trial Questionnaire (EU-Dap) for substance use screening and to assess risk and protective factors among early adolescents in Chile
Source: PLoS One. 2021 Oct 11;16(10):e0258288. doi: 10.1371/journal.pone.0258288 (PMC8504767; doi:10.1371/journal.pone.0258288)
Supplement: S2 Questionnaire — (DOCX) [file pone.0258288.s002.docx]

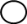

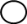

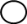

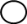

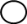

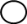

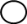

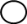

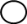

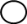

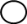

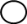

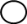

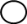

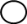

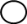

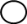

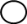

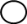

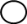

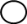

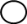

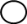

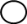

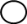

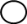

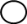

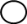

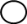

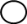

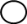

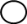

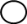

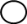

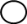

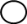

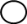

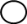

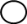

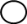

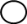

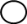

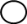

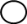

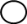

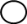

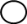

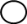

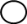

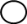

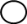

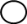

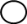

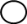

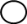

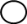

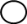

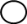

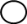

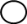

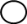

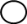

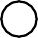

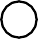

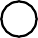

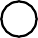


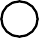

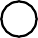


ID:

Cuestionario EU-DAP

| **USO INTERNO** |
| --- |
| Nombre del evaluador: |
| Establecimiento educacional: |
| RUT: |
| Fecha de hoy: |

| Nombre completo: | | | | |
| --- | --- | --- | --- | --- |
| Edad: | | | | |
| En qué país naciste: |  |  |  |  |
| Chile Venezuela Colombia Haití  Perú Bolivia  Otro: | | | | |
| ¿En qué día naciste? |  |  |  |  |
| 1 | 8 | 14 | 20 | 26 |
| 2 | 9 | 15 | 21 | 27 |
| 3 | 10 | 16 | 22 | 28 |
| 4 | 11 | 17 | 23 | 29 |
| 5 | 12 | 18 | 24 | 30 |
| 6 | 13 | 19 | 25 | 31 |
| 7 |  |  |  |  |
| ¿En qué mes naciste?: |  | |  | |
| Enero | Mayo | | Septiembre | |
| Febrero | Junio | | Octubre | |
| Marzo | Julio | | Noviembre | |
| Abril | Agosto | | Diciembre | |
| ¿En qué año naciste? |  | |  | |
| 2001 | 2005 | | 2009 | |
| 2002 | 2006 | | 2010 | |
| 2003 | 2007 | | 2011 | |
| 2004 | 2008 | | 2012 | |

| Curso: | 5º | 6º | 7º | 8º |  |
| --- | --- | --- | --- | --- | --- |
| Letra del curso: | A | B | C | D | E |

1. Sexo


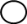


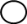


Hombre Mujer

1. ¿Cuáles de las siguientes personas viven contigo en tu casa? Marca todas las respuestas necesarias.

|  | Sí | No |
| --- | --- | --- |
| a) Padre | O | O |
| b) Padrastro | O | O |
| c) Madre | O | O |
| d) Madrastra | O | O |
| e) Hermano/s y/o hermanastro/a | O | O |
| f) Abuelo/s | O | O |
| g) Otro/s pariente/s | O | O |
| h) No pariente/s | O | O |

1. ¿Tienes hermanos, incluyendo a hermanastros?

|  | Sí | No |
| --- | --- | --- |
| a) Hermano/s mayor/es | O | O |
| b) Hermano/s menor/es | O | O |
| c) Gemelo o mellizo | O | O |

1.
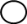
¿Cuántas veces, si es que lo has hecho, has fumado cigarrillos **EN TODA TU VIDA**? 0 veces


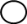
1 – 2 veces


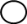
3 – 5 veces


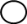
6 – 9 veces


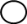
10 – 19 veces


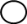

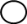
20 – 29 veces 30 o más veces

1. ¿Cuántas veces, si es que lo has hecho, has fumado cigarrillos **EN LOS ÚLTIMOS 12 MESES**?
2.
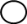
¿Cuántas veces, si es que lo has hecho, has fumado cigarrillos **EN LOS ÚLTIMOS 30 DÍAS**? 0 veces


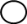
1 – 2 veces


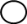
3 – 5 veces


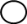
6 – 9 veces


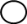
10 – 19 veces


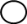

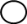
20 – 29 veces 30 o más veces

1. ¿Cuántos cigarrillos sueles fumar a la semana? Si fumas menos de una vez a la semana o si no fumas, por favor marca el 0.


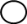
0


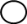
1 – 2


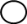
3 – 5


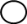
6 – 9


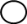
10 – 19


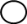
20 o más

1. ¿Qué probabilidad hay de que te ocurran las siguientes cosas si fumaras cigarrillos el mes que viene? Marca la respuesta más cercana a tu opinión.

|  | Muy probable | Probable | Poco  probable | Muy poco  probable |
| --- | --- | --- | --- | --- |
| a) Meterme en problemas con mis padres | O | O | O | O |
| b) Tener problemas con mis amigos/as | O | O | O | O |
| c) Volverme adicto/a | O | O | O | O |
| d) Tener problemas de dinero | O | O | O | O |
| e) Sentirme más relajado/a | O | O | O | O |
| f) Divertirme más | O | O | O | O |
| g) Ser más popular | O | O | O | O |
| h) Sentirme más seguro/a de ti mismo/a | O | O | O | O |

1. ¿Cuántas veces, si es que ha ocurrido, has consumido bebidas alcohólicas **EN TODA TU VIDA**?
2. ¿Cuantas veces, si es que ha ocurrido, has consumido bebidas alcohólicas **EN LOS ÚLTIMOS 12 MESES**?
3.
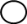
veces
4.
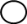
– 2 veces


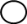
3 – 5 veces


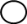
6 – 9 veces


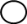
10 – 19 veces


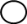

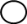
20 – 29 veces 30 o más veces

1. ¿Cuantas veces, si es que ha ocurrido, has consumido bebidas alcohólicas **EN LOS ÚLTIMOS 30 DÍAS**?
2.
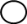
veces
3.
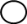
– 2 veces


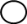
3 – 5 veces

6 – 9 veces

10 – 19 veces

20 – 29 veces 30 o más veces

1. Actualmente, ¿Cada cuánto tiempo tomas bebidas alcohólicas, por ejemplo, cerveza, vino o destilados? Trata de incluir incluso esas veces en que sólo tomas una pequeña cantidad.

Todos los días Todas las semanas Todos los meses Rara vez

Nunca

1. ¿Cuántas veces, si es que ha ocurrido, te has emborrachado por tomar bebidas alcohólicas

# EN TODA TU VIDA?

1. veces
2. – 2 veces

3 – 5 veces

6 – 9 veces

10 – 19 veces

20 – 29 veces 30 o más veces

1. ¿Cuántas veces, si es que ha ocurrido, te has emborrachado por tomar bebidas alcohólicas

# EN LOS ÚLTIMOS 12 MESES?

1. veces
2. – 2 veces

3 – 5 veces

6 – 9 veces

10 – 19 veces

20 – 29 veces 30 o más veces

1. ¿Cuántas veces, si es que ha ocurrido, te has emborrachado por tomar bebidas alcohólicas

# EN LOS ÚLTIMOS 30 DÍAS?

1. veces
2. – 2 veces

3 – 5 veces

6 – 9 veces

10 – 19 veces

20 – 29 veces 30 o más veces

1. ¿Cuántas veces, si es que lo has hecho, has tomado 4 tragos en un par de horas? 0 veces

1 – 2 veces

3 – 5 veces

6 – 9 veces

10 – 19 veces

20 – 29 veces 30 veces o más

1. ¿Cuántas veces, si es que lo has hecho, has tomado 5 tragos en un par de horas? 0 veces

1 – 2 veces

3 – 5 veces

6 – 9 veces

10 – 19 veces

20 – 29 veces 30 veces o más

1. ¿Qué probabilidad hay de que te ocurran las siguientes cosas si tomas alcohol el mes que viene? Marca la respuesta más cercana a tu opinión

|  | Muy  probable | Probable | Poco  probable | Muy poco  probable |
| --- | --- | --- | --- | --- |
| a) Que me vaya mal en el colegio | O | O | O | O |
| b) Meterme en problemas con mis padres | O | O | O | O |
| c) Tener problemas con mis amigos/as | O | O | O | O |
| d) Volverme adicto/a | O | O | O | O |
| e) Tener problemas de dinero | O | O | O | O |
| f) Sentirme más relajado/a | O | O | O | O |
| g) Divertirme más | O | O | O | O |
| h) Ser más popular | O | O | O | O |
| i) Olvidar mis problemas | O | O | O | O |
| j) Sentirme más seguro/a de ti mismo/a | O | O | O | O |

1. ¿Cuántas veces, si es que lo has hecho, has consumido marihuana **EN TODA TU VIDA**? 0 veces

1 – 2 veces

3 – 5 veces

6 – 9 veces

10 – 19 veces

20 – 29 veces 30 o más veces

1. ¿Cuántas veces, si es que lo has hecho, has consumido marihuana **EN LOS ÚLTIMOS 12 MESES**?
2. veces
3. – 2 veces

3 – 5 veces

6 – 9 veces

10 – 19 veces

20 – 29 veces 30 o más veces

1. ¿Cuántas veces, si es que lo has hecho, has consumido marihuana **EN LOS ÚLTIMOS 30 DÍAS**?
2. veces
3. – 2 veces

3 – 5 veces

6 – 9 veces

10 – 19 veces

20 – 29 veces 30 o más veces

1. ¿Qué probabilidad hay que te ocurran estas cosas si consumes marihuana el mes que viene?

|  | Muy probable | Probable | Poco probable | Muy poco probable |
| --- | --- | --- | --- | --- |
| a) Meterme en problemas con la policía | O | O | O | O |
| b) Tener problemas en el colegio | O | O | O | O |
| c) Meterme en problemas con mis padres | O | O | O | O |
| d) Tener problemas con mis amigos/as | O | O | O | O |
| e) Volverme adicto/a | O | O | O | O |
| f) Tener problemas de dinero | O | O | O | O |
| g) Sentirme más relajado/a | O | O | O | O |
| h) Divertirme más | O | O | O | O |
| i) Ser más popular | O | O | O | O |
| j) Sentirme más seguro/a de ti mismo/a | O | O | O | O |

1. ¿Has consumido alguna vez alguna de las siguientes drogas? Marca una o más casillas en cada línea.

|  | No | Sí, en los últimos 30  días | Sí, en los últimos  12 meses | Sí, a veces en  mi vida |
| --- | --- | --- | --- | --- |
| a) Tranquilizantes o sedantes (sin receta médica) | O | O | O | O |
| b) LSD u otros alucinógenos | O | O | O | O |
| c) Anfetaminas | O | O | O | O |
| d) Cocaína | O | O | O | O |
| e) Heroína | O | O | O | O |
| f) Éxtasis | O | O | O | O |
| g) Pasta Base | O | O | O | O |
| h) Inhalantes | O | O | O | O |

1. ¿Qué probabilidad hay de que hagas las siguientes cosas DE AQUÍ A UN AÑO? Marca una casilla en cada línea.

| Muy  probable | | Probable | Poco  probable | Muy poco  probable |
| --- | --- | --- | --- | --- |
| a) Fumar cigarrillos | O | O | O | O |
| b) Tomar bebidas alcohólicas (cerveza, vino,  destilados) | O | O | O | O |
| c) Emborracharme | O | O | O | O |
| d) Fumar marihuana | O | O | O | O |
| e) Inhalar sustancias (pegamento u otros) | O | O | O | O |
| f) Tomar sustancias ilegales | O | O | O | O |

1. A continuación, hay varias afirmaciones que la gente ha hecho sobre sustancias ilegales

¿Estás de acuerdo con las siguientes opiniones sobre drogas? Marca la respuesta más cercana a tu opinión.

|  | Muy de  acuerdo | De acuerdo | En  desacuerdo | Muy en  desacuerdo |
| --- | --- | --- | --- | --- |
| a) Consumir drogas puede ser una  actividad agradable | O | O | O | O |
| b) Una persona joven (menor de 18  años) nunca debería probar drogas | O | O | O | O |
| c) Consumir dogas es divertido | O | O | O | O |
| d) Hay muchas cosas más peligrosas que  probar drogas | O | O | O | O |
| e) Todo el mundo que prueba las drogas  se arrepiente con el tiempo | O | O | O | O |
| f) Las leyes sobre drogas deberían ser  más duras | O | O | O | O |
| g) El consumo de drogas es uno de los  mayores males del país | O | O | O | O |
| h) Las drogas ayudan a que la gente  tenga una vida completamente feliz | O | O | O | O |
| i) Las escuelas deberían explicar los verdaderos peligros de consumir  drogas | O | O | O | O |
| j) La policía no debería molestar a la  gente joven que está probando drogas | O | O | O | O |
| k) Experimentar con drogas es perder el  control de tu vida | O | O | O | O |

1. A continuación, en cada afirmación, por favor marca si crees que es correcta o no en el círculo que corresponda.

|  | Sí | No | No lo  sé |
| --- | --- | --- | --- |
| a) La nicotina es la sustancia de los cigarrillos que causa cáncer de  pulmón | O | O | O |
| b) Hay que fumar varios cigarrillos al día, durante muchos años, para volverse adicto | O | O | O |
| c) Las mujeres tienen menor tolerancia al alcohol que los hombres | O | O | O |
| d) Lleva una media hora eliminar del cuerpo la cantidad de alcohol  que contiene una lata de cerveza fuerte | O | O | O |
| e) Fumar marihuana no causa dependencia física (dependencia física  es la necesidad de consumir cada vez más) | O | O | O |
| f) Un alto consumo de marihuana disminuye la producción de  hormonas sexuales | O | O | O |

1. ¿Cuánto crees que SE ARRIESGA LA GENTE a dañarse (físicamente o de otras maneras), si…?

Marca una casilla en cada línea.

|  | Ningún riesgo | Riesgo leve | Gran riesgo | No sé |
| --- | --- | --- | --- | --- |
| a) Fuma cigarros de forma ocasional | O | O | O | O |
| b) Fuma una o más cajetillas de cigarros al día | O | O | O | O |
| c) Toma una o dos bebidas alcohólicas casi todas las  semanas | O | O | O | O |
| d) Toma alcohol todos los días | O | O | O | O |
| e) Prueba sustancias inhalantes (pegamento u otros)  una o dos veces | O | O | O | O |
| f) Prueba marihuana una o dos veces | O | O | O | O |
| g) Fuma marihuana de forma regular | O | O | O | O |
| h) Usa otras drogas ocasionalmente | O | O | O | O |

1. ¿Alguna de las siguientes personas fuma cigarrillos?

|  | Fuma a diario | Fuma a veces | No fuma | No sé | No aplica, no tengo padre/madre vivo, o no lo/a veo nunca |
| --- | --- | --- | --- | --- | --- |
| a) Madre | O | O | O | O | O |
| b) Padre | O | O | O | O | O |
| c) Mejor/es  amigos/as | O | O | O | O | O |
| d) Hermanos/as | O | O | O | O | O |

1. Cuando respondas esta pregunta, piensa en los amigos/as con quienes pasas la mayor parte de tu tiempo libre. Marca una casilla en cada línea.

|  | Ninguno | Menos  de la mitad | Sobre  la mitad | Más de  la mitad | Todos ellos | No lo sé |
| --- | --- | --- | --- | --- | --- | --- |
| a) ¿A cuántos les gusta el colegio? | O | O | O | O | O | O |
| b) ¿Cuántos se sacan buenas notas? | O | O | O | O | O | O |
| c) ¿Cuántos fuman cigarrillos? | O | O | O | O | O | O |
| d) ¿Cuántos se emborrachan? | O | O | O | O | O | O |
| e) ¿Cuántos consumen marihuana u  otras drogas? | O | O | O | O | O | O |

1. ¿Alguno de tus hermanos/as…? Marca una casilla en cada línea.

|  | Sí | No | No lo sé | No tengo hermanos |
| --- | --- | --- | --- | --- |
| a) Toma bebidas alcohólicas (cerveza, vino,  destilados) | O | O | O | O |
| b) Se emborracha | O | O | O | O |
| c) Fuma marihuana | O | O | O | O |
| d) Inhala alguna sustancia (pegamento, bencina) | O | O | O | O |
| e) Toma otras drogas | O | O | O | O |

1. ¿Alguna de las siguientes descripciones se adapta a la gente a tu alrededor? Marca la respuesta más cercana a tu opinión.

|  | Muy de acuerdo | De acuerdo | En desacuerdo | Muy en desacuerdo |
| --- | --- | --- | --- | --- |
| a) Mis padres establecen normas claras | O | O | O | O |
| b) Mis padres saben dónde estoy por las  tardes | O | O | O | O |
| c) Puedo conseguir fácilmente el apoyo de  mi padre y/o madre | O | O | O | O |
| d) Para mí es muy importante no defraudar  o decepcionar a mis padres | O | O | O | O |
| e) Mi/s mejor/es amigos/as me apoya de  verdad | O | O | O | O |

1. En las próximas preguntas, vas a decir si estás de acuerdo o no con cada afirmación sobre tu familia. Marca la respuesta más cercana a tu opinión.

| Totalmente en  desacuerdo | | En desacuerdo | De acuerdo | Totalmente de acuerdo |
| --- | --- | --- | --- | --- |
| a) En mi familia realmente nos ayudamos y  apoyamos los unos a los otros | O | O | O | O |
| b) En mi familia no se discuten los  problemas | O | O | O | O |
| c) No nos solemos pelear en mi familia | O | O | O | O |
| d) Las tareas de cada persona están  claramente establecidas en mi familia | O | O | O | O |
| e) En mi familia puedes salirte con la tuya  con casi cualquier cosa. | O | O | O | O |
| f) En mi familia todos estamos llenos de vida  y buen humor | O | O | O | O |
| g) En mi familia a todos nos importa  expresar nuestra propia opinión | O | O | O | O |
| h) En mi familia rara vez perdemos el  control | O | O | O | O |
| i) Hay un castigo estricto para cualquiera  que rompa las normas en mi familia | O | O | O | O |
| j) En mi familia cada uno hace lo que quiere | O | O | O | O |
| k) En mi familia siempre hacemos cosas  juntos | O | O | O | O |

| Totalmente en  desacuerdo | | En desacuerdo | De acuerdo | Totalmente de acuerdo |
| --- | --- | --- | --- | --- |
| L) Hay muchas discusiones en mi familia | O | O | O | O |
| m) En mi familia nunca nos pegamos los  unos a los otros | O | O | O | O |
| n) “Hacer las tareas antes de jugar” es la  norma en mi familia | O | O | O | O |
| o) En mi familia no se nos castiga o reta  cuando hacemos algo mal | O | O | O | O |
| P) Nos llevamos realmente bien los unos con  los otros | O | O | O | O |
| q) No nos contamos nuestros problemas  personales | O | O | O | O |
| r) En mi familia no solemos criticar a los  demás | O | O | O | O |
| s) Los miembros de mi familia tienen ideas estrictas sobre lo que está bien y lo que está  mal | O | O | O | O |
| t) En mi familia entramos y salimos de la  casa cuando queremos | O | O | O | O |

1. ¿Si quisieras tomar alcohol (o si ya lo quieres), crees que tu padre y madre te lo permitirían? Marca una casilla en cada línea.

|  | Me permitiría (me permite) tomar alcohol | No me permitiría (no me permite) beber en casa | No me permitiría (no me permite) tomar de ninguna manera | No lo sé | No aplica, no tengo padre/madre vivo, o no lo/a veo  nunca |
| --- | --- | --- | --- | --- | --- |
| a) Padre | O | O | O | O | O |
| b) Madre | O | O | O | O | O |

1. ¿Si quisieras fumar cigarros (o si ya quieres), crees que tu padre y madre te lo permitirían? Marca una casilla en cada línea.

| Me permitiría (me permite) fumar cigarros | | No me permitiría (no me permite) fumar cigarros  en casa | No me permitiría (no me permite) fumar cigarros de ninguna manera | No lo sé | No aplica, no tengo padre/madre vivo, o no lo/a  veo nunca |
| --- | --- | --- | --- | --- | --- |
| a) Padre | O | O | O | O | O |
| b) Madre | O | O | O | O | O |

1. ¿Si quisieras consumir marihuana (o si ya quieres), crees que tu padre y madre te lo permitirían? Marca una casilla en cada línea.

| Me permitiría (me permite) consumir marihuana | | No me permitiría (no me permite) consumir marihuana en  casa | No me permitiría (no me permite) consumir marihuana de ninguna manera | No lo sé | No aplica, no tengo padre/madre vivo, o no lo/a veo nunca |
| --- | --- | --- | --- | --- | --- |
| a) Padre | O | O | O | O | O |
| b) Madre | O | O | O | O | O |

1. ¿Cómo fueron tus notas el año pasado comparadas con las de tus compañeros de curso? Mucho mejores

Mejores

Como las de la mayoría Peores

1. En tu opinión ¿mejorarán tus notas al final de este año? Sí

Probablemente sí Probablemente no No

1. ¿Qué opinas de tu escuela actualmente? Me gusta mucho

Me gusta un poco No me gusta mucho No me gusta nada

1. ¿Estás de acuerdo con las siguientes descripciones de tu colegio? Marca una casilla en cada línea.

|  | Muy de  acuerdo | De  acuerdo | En  desacuerdo | Muy en  desacuerdo |
| --- | --- | --- | --- | --- |
| a) A los estudiantes de mi curso les gusta estar juntos | O | O | O | O |
| b) La mayoría de la gente de mi curso es  amable y te ayuda | O | O | O | O |
| c) Los demás estudiantes me aceptan como  soy | O | O | O | O |
| d) Me preocupa mucho cómo me va en el  colegio | O | O | O | O |
| e) Respeto mucho lo que me dicen los  profesores | O | O | O | O |

1. ¿Has tenido alguno de los siguientes problemas en los últimos 12 meses? Marca todas las respuestas necesarias en cada línea.

|  | **Nunca** | **Sí, por tomar alcohol** | **Sí, por consumir drogas** | **Sí, por razones distintas a tomar alcohol o consumir**  **drogas** |
| --- | --- | --- | --- | --- |
| a) Discusión | O | O | O | O |
| b) Pelea | O | O | O | O |
| c) Accidente o herida | O | O | O | O |
| d) Pérdida de dinero o de  objetos valiosos | O | O | O | O |
| e) Daños a objetos o ropa  que te pertenecía | O | O | O | O |
| f) Problemas en la relación  con (tus) padres | O | O | O | O |
| g) Problemas en la relación  con (tus) amigos/as | O | O | O | O |
| h) Problemas en la relación  con (tus) profesores | O | O | O | O |
| i) Malas notas en la escuela | O | O | O | O |
| j) Víctima de robo o hurto | O | O | O | O |
| k) Hospitalizado o llevado a  urgencias | O | O | O | O |

1. Hay varias formas posibles de tomar decisiones ¿Cómo se adaptan a ti las siguientes? Marca la respuesta más cerca de tu opinión.

|  | Muy de  acuerdo | De  acuerdo | En  desacuerdo | Muy en  desacuerdo |
| --- | --- | --- | --- | --- |
| a) Cuando decido hacer algo, siempre lo llevo  a cabo | O | O | O | O |
| b) Suelo tomar decisiones sin pensar en las  consecuencias | O | O | O | O |
| c) Evalúo todas las opciones antes de  decidirme por algo | O | O | O | O |
| d) Suelo arrepentirme de lo que he decidido | O | O | O | O |
| e) Cuando decido algo, no me importa lo que  piensen mis amigos/as | O | O | O | O |

1. Imagínate en cada una de las siguientes situaciones. Algunas te pueden resultar muy familiares, otras menos, por lo que puedes sentirte menos seguro al contestar. Es suficiente con que hagas lo que puedas. Marca la respuesta más cercana a tu opinión.

|  | Muy  probable | Probable | Improbable | Muy  improbable |
| --- | --- | --- | --- | --- |
| a) Estás con tu mejor amigo/a en una fiesta en la que puedes conocer a gente nueva. Tú realmente quieres conocerlos. Alguien te ofrece fumar marihuana juntos. Tu  amigo/a acepta. ¿Y tú? | O | O | O | O |
| b) Estás con el mismo amigo/a estudiando para un examen importante que tienen al día siguiente. Los dos están estresados y necesitan calmarse. Tu amigo/a sugiere que un cigarro podría ayudar y te ofrece  uno. ¿Aceptas? | O | O | O | O |
| c) Al día siguiente, los dos aprueban el examen y creen que hay que celebrarlo. Les queda algo de dinero y la botillería está al lado. ¿Comprarían algo de alcohol  (cerveza, vino, destilados) para celebrar? | O | O | O | O |

1. ¿Estás de acuerdo con las siguientes descripciones de ti mismo/a? Marca la respuesta más cercana a tu opinión.

|  | Muy de  acuerdo | De acuerdo | En  desacuerdo | Muy en  desacuerdo |
| --- | --- | --- | --- | --- |
| a) Creo que tengo muchas buenas  cualidades | O | O | O | O |
| b) Puedo hacer las cosas tan bien como  la mayoría de la gente | O | O | O | O |
| c) A veces pienso que no soy bueno/a  en nada | O | O | O | O |
| d) La mayoría de la gente de mi edad es  más inteligente que yo | O | O | O | O |
| e) Soy bastante bueno/a en deporte | O | O | O | O |
| f) Me da vergüenza cuando tengo que  decir algo en clases | O | O | O | O |
| g) Para mis padres es importante que  yo esté feliz | O | O | O | O |
| h) Me preocupo mucho por cosas  tontas | O | O | O | O |
| i) No me suelo poner nervioso/a por  nada | O | O | O | O |
| j) Tengo muchos intereses y  pasatiempos | O | O | O | O |

1. A continuación hay varias afirmaciones sobre las relaciones con otras personas. Marca la respuesta más cercana a tu opinión.

|  | Muy de  acuerdo | De  acuerdo | En  desacuerdo | Muy en  desacuerdo |
| --- | --- | --- | --- | --- |
| a) Cuando intentan humillarte,  deberías hacerles lo mismo | O | O | O | O |
| b) Vale la pena que la gente sepa que  estás enfadado con ellos | O | O | O | O |
| c) La única forma de tratar con los agresores es hacerles saber quién  manda | O | O | O | O |
| d) Siempre hay formas de arreglar los  problemas sin tener que pelear | O | O | O | O |
| e) Es mucho mejor perder el control  (enojarse mucho) que explicar las cosas tranquilamente | O | O | O | O |

1. Imagina que quisieras hacer las siguientes cosas. ¿Qué tan fácil o difícil te resultaría? Marca una casilla en cada línea.

Me gustaría…

|  | **Muy**  **fácil** | **Fácil** | **Difícil** | **Muy**  **difícil** |
| --- | --- | --- | --- | --- |
| a) Decir algo agradable a un amigo/a | O | O | O | O |
| b) Pedir un favor | O | O | O | O |
| c) Mostrarle a alguien que me gusta | O | O | O | O |
| d) Decir “no” cuando me piden que haga algo  que no quiero hacer | O | O | O | O |
| e) Pedir ayuda cuando tengo problemas | O | O | O | O |
| f) Ayudar a alguien que necesita ayuda | O | O | O | O |

Reprinted with permission from Federica Vigna-Taglianti, originally published in [2003].
