# Supplementary material for: Validation of the European Drug Addiction Prevention Trial Questionnaire (EU-Dap) for substance use screening and to assess risk and protective factors among early adolescents in Chile
Source: PLoS One. 2021 Oct 11;16(10):e0258288. doi: 10.1371/journal.pone.0258288 (PMC8504767; doi:10.1371/journal.pone.0258288)
Supplement: S1 Table — (DOCX) [file pone.0258288.s003.docx]

S1 Table. Family structure

| Variables | n | % | [95% CI] |
| --- | --- | --- | --- |
| Family Structure |  |  |  |
| Lives with father | 1416 | 69.6 | [67.5-71.5] |
| Lives with mother | 2049 | 94.0 | [92.9-94.9] |
| Lives with siblings | 1831 | 87.3 | [85.8-88.6] |
| Has older siblings | 1418 | 73.7 | [71.7-75.7] |
| Has younger siblings | 1254 | 69.0 | [66.8-71.1] |
| Has a twin | 80 | 5.8 | [4.7-7.1] |
| Lives with grandparents | 584 | 33.6 | [31.4-35.8] |
| Lives with stepfather | 293 | 17.3 | [15.5-19.1] |
| Lives with stepmother | 69 | 4.3 | [3.4-5-4] |
| Lives with other relatives | 495 | 29.2 | [27.1-31.4] |
| Lives with non-relatives | 268 | 17.3 | [15.5-19.3] |
