# Supplementary material for: Validation of the European Drug Addiction Prevention Trial Questionnaire (EU-Dap) for substance use screening and to assess risk and protective factors among early adolescents in Chile
Source: PLoS One. 2021 Oct 11;16(10):e0258288. doi: 10.1371/journal.pone.0258288 (PMC8504767; doi:10.1371/journal.pone.0258288)
Supplement: S2 Table — (DOCX) [file pone.0258288.s004.docx]

S2 Table. Description of scales

| **Scales and Questions** | **Options** | **Item number** | **n** | **Mean** | **SD** | **Kurtosis** | **Skewness** | **Factor Loading** |
| --- | --- | --- | --- | --- | --- | --- | --- | --- |
| **Positive and negative beliefs about tobacco use**  How likely is that each of the following would happen to you if you smoke cigarettes in  the next month? Mark the answer that is closest to your opinion. | *Positive beliefs* |  |  |  |  |  |  |  |
|  | Feel more relaxed | 5 | 2067 | 2.91 | 1.08 | 1.88 | -0.47 | 0.68 |
|  | Have more fun | 6 | 2067 | 3.16 | 1.01 | 2.59 | -0.91 | 0.88 |
|  | Be more popular | 7 | 2067 | 3.37 | 0.92 | 3.61 | -1.32 | 0.67 |
|  | Be more confident and outgoing | 8 | 2067 | 3.49 | 0.89 | 4.81 | -1.71 | 0.85 |
|  | *Negative beliefs* |  |  |  |  |  |  |  |
|  | Get into trouble with parents | 1 | 2067 | 1.63 | 1.02 | 3.69 | 1.45 | 0.73 |
|  | Have problems with my friends | 2 | 2067 | 2.69 | 1.04 | 1.85 | -0.20 | 0.59 |
|  | Become an addict | 3 | 2067 | 2.37 | 1.24 | 1.42 | 0.18 | 0.79 |
|  | Have money problems | 4 | 2067 | 2.82 | 1.15 | 1.70 | -0.41 | 0.70 |
| **Positive and negative beliefs about alcohol use**  How likely is that each of the following would happen to you if you drink alcohol in the  next month? Mark the answer that is closest to your opinion. | *Positive beliefs* |  |  |  |  |  |  |  |
|  | Feel more relaxed | 6 | 2046 | 3.11 | 1.02 | 2.39 | -0.81 | 0.81 |
|  | Have more fun | 7 | 2046 | 3.07 | 1.04 | 2.22 | -0.73 | 0.91 |
|  | Be more popular | 8 | 2046 | 3.38 | 0.91 | 3.71 | -1.33 | 0.73 |
|  | Forget my troubles | 9 | 2046 | 2.92 | 1.13 | 1.81 | -0.52 | 0.76 |
|  | Be more confident and outgoing | 10 | 2046 | 3.47 | 0.92 | 4.58 | -1.66 | 0.83 |
|  | *Negative beliefs* |  |  |  |  |  |  |  |
|  | Do badly in school | 1 | 2046 | 2.06 | 1.10 | 1.98 | 0.60 | 0.86 |
|  | Get into trouble with parents | 2 | 2046 | 1.64 | 1.02 | 3.55 | 1.40 | 0.84 |
|  | Have problems with my friends | 3 | 2046 | 2.53 | 1.09 | 1.70 | -0.02 | 0.74 |
|  | Become an addict | 4 | 2046 | 2.42 | 1.22 | 1.43 | 0.11 | 0.80 |
|  | Have money problems | 5 | 2046 | 2.83 | 1.14 | 1.74 | -0.44 | 0.72 |
| **Positive and negative**  **beliefs about marijuana**  **use**  How likely is that each of the following would happen to you if you take marijuana or  other illegal substances in the next month? Mark the answer that is closest to your opinion. | *Positive beliefs* |  |  |  |  |  |  |  |
|  | Feel more relaxed | 7 | 2101 | 2.68 | 1.21 | 1.48 | -0.20 | 0.81 |
|  | Have more fun | 8 | 2101 | 2.97 | 1.13 | 1.91 | -0.61 | 0.95 |
|  | Be more popular | 9 | 2101 | 3.36 | 0.95 | 3.59 | -1.34 | 0.73 |
|  | Be more confident and outgoing | 10 | 2101 | 3.42 | 0.95 | 4.16 | -1.55 | 0.85 |
|  | *Negative beliefs* |  |  |  |  |  |  |  |
|  | Get into trouble with police | 1 | 2101 | 1.85 | 1.09 | 2.45 | 0.95 | 0.88 |
|  | Have problems in school | 2 | 2101 | 1.78 | 1.04 | 2.90 | 1.10 | 0.94 |
|  | Get into trouble with parents | 3 | 2101 | 1.49 | 0.95 | 5.02 | 1.85 | 0.92 |
|  | Have problems with my friends | 4 | 2101 | 2.28 | 1.14 | 1.69 | 0.31 | 0.77 |
|  | Become an addict | 5 | 2101 | 2.12 | 1.22 | 1.66 | 0.53 | 0.77 |
|  | Have money problems | 6 | 2101 | 2.55 | 1.21 | 1.46 | -0.06 | 0.69 |
| **Future substance use**    How likely is it that you will be doing each of the following a year from now? Mark one box for each line. | Smoke cigarettes | 1 | 2165 | 3.66 | 0.73 | 7.66 | -2.30 | 0.88 |
|  | Drink alcoholic beverages (beer, wine, spirits) | 2 | 2165 | 3.40 | 0.90 | 3.67 | -1.34 | 0.83 |
|  | Get drunk | 3 | 2165 | 3.69 | 0.71 | 8.56 | -2.48 | 0.90 |
|  | Smoke marijuana or hashish (pot, grass) | 4 | 2165 | 3.76 | 0.66 | 11.67 | -3.03 | 0.90 |
|  | Sniff a substance (glue etc.) to get high. | 5 | 2165 | 3.83 | 0.57 | 17.17 | -3.77 | 0.82 |
|  | Take illegal substances | 6 | 2165 | 3.85 | 0.54 | 19.48 | -4.04 | 0.93 |
| **Positive and negative attitudes towards drugs**  Here are some statements that people have made about illegal substances.  How much do you agree with the following opinions on drugs?  Mark the answer that is closest to your opinion. | *Positive beliefs* |  |  |  |  |  |  |  |
|  | Using drugs can be a pleasant activity | 1 | 2039 | 3.43 | 0.82 | 4.11 | -1.37 | 0.77 |
|  | Using drugs is fun | 3 | 2039 | 3.61 | 0.67 | 5.95 | -1.78 | 0.88 |
|  | The police should not be annoying young people who are trying drugs | 10 | 2039 | 3.52 | 0.85 | 5.37 | -1.82 | 0.68 |
|  | Drugs help people to have experience life in full | 8 | 2039 | 3.60 | 0.71 | 6.57 | -1.93 | 0.76 |
|  | *Negative beliefs* |  |  |  |  |  |  |  |
|  | A young person should never try drugs | 2 | 2039 | 1.83 | 1.13 | 2.42 | 0.99 | 0.68 |
|  | Everyone who tries drugs eventually regrets it | 5 | 2039 | 2.24 | 1.06 | 1.87 | 0.33 | 0.61 |
|  | The laws about drugs should be made stronger | 6 | 2039 | 1.78 | 1.00 | 2.91 | 1.07 | 0.83 |
|  | Drug use is one of the biggest evils in the country | 7 | 2039 | 1.86 | 1.01 | 2.64 | 0.90 | 0.81 |
|  | Many things are much more risky than trying drugs | 4 | 2039 | 2.43 | 1.07 | 1.79 | 0.16 | 0.49 |
|  | Schools should teach about the real hazards of taking drugs | 9 | 2039 | 1.54 | 0.92 | 4.75 | 1.70 | 0.81 |
|  | To experiment with drugs is to give away control of your life | 11 | 2039 | 1.92 | 1.09 | 2.27 | 0.82 | 0.72 |
| **Risk perception**  How much do you think people risk (physically or in other ways), if they… Mark one box for each line. | Smoke cigarettes occasionally | 1 | 1199 | 1.26 | 0.59 | 2.48 | -0.14 | 0.65 |
|  | Smoke one or more packs of cigarettes per day | 2 | 1199 | 1.86 | 0.41 | 12.29 | -3.11 | 0.78 |
|  | Have one or two drinks nearly each week | 3 | 1199 | 1.31 | 0.62 | 2.34 | -0.32 | 0.57 |
|  | Drink alcohol every day | 4 | 1199 | 1.88 | 0.39 | 15.09 | -3.53 | 0.83 |
|  | Try inhalants (glue etc.) once or twice | 5 | 1199 | 1.58 | 0.59 | 3.15 | -1.08 | 0.65 |
|  | Try marijuana or hashish (cannabis, pot, grass) once or twice | 6 | 1199 | 1.34 | 0.63 | 2.32 | -0.40 | 0.71 |
|  | Smoke marijuana or hashish regularly | 7 | 1199 | 1.67 | 0.57 | 4.38 | -1.54 | 0.68 |
|  | Use other drugs occasionally | 8 | 1199 | 1.72 | 0.53 | 5.02 | -1.71 | 0.78 |
| **Normative beliefs**  When you answer this question, think about the friends with whom you spend most of  your leisure time. Mark one box for each line. | How many of them like school? | 1 | 1191 | 1.71 | 1.26 | 2.10 | 0.40 | -0.05 |
|  | How many of them do well at school? | 2 | 1191 | 1.98 | 0.99 | 2.36 | 0.24 | -0.15 |
|  | How many of them smoke cigarettes? | 3 | 1191 | 0.20 | 0.55 | 19.00 | 3.62 | 0.95 |
|  | How many of them get drunk? | 4 | 1191 | 0.22 | 0.60 | 18.19 | 3.60 | 0.88 |
|  | How many of them use marijuana or other drugs? | 5 | 1191 | 0.18 | 0.55 | 23.27 | 4.14 | 0.94 |
| **Parenting Involvement**  Do the following descriptions fit people around you? Mark the answer that is closest to your opinion. | My parents set clear rules | 1 | 2101 | 1.48 | 0.71 | 5.65 | 1.62 | 0.76 |
|  | My parents know where I am in the evenings | 2 | 2101 | 1.39 | 0.70 | 6.78 | 1.99 | 0.80 |
|  | I can easily get support from my father and/or mother | 3 | 2101 | 1.46 | 0.76 | 5.43 | 1.71 | 0.74 |
|  | It is very important for me not to disappoint my parents | 4 | 2101 | 1.42 | 0.72 | 6.21 | 1.86 | 0.74 |
|  | I can really get support from my best friend | 5 | 2101 | 1.55 | 0.77 | 4.60 | 1.43 | 0.54 |
| **Family functioning**  In the following questions, you are to say whether you agree or disagree with each statement about your family. Mark the answer that is closest to your opinion. | In my family we really help and support one another | 1 | 1848 | 3.38 | 0.86 | 4.29 | -1.41 | 0.80 |
|  | My family does not discuss its problems | 2 | 1848 | 2.16 | 0.94 | 2.28 | 0.42 | 0.48 |
|  | We don’t often fight in my family | 3 | 1848 | 2.57 | 0.96 | 2.06 | -0.05 | 0.55 |
|  | Each person’s duties are clearly set out in my family | 4 | 1848 | 2.90 | 0.93 | 2.31 | -0.45 | 0.67 |
|  | In my family you can get away with almost anything | 5 | 1848 | 1.77 | 0.84 | 3.50 | 1.00 | 0.65 |
|  | In my family we are full of life and good spirits | 6 | 1848 | 3.12 | 0.89 | 2.93 | -0.82 | 0.83 |
|  | In my family it’s important for everyone to express their own opinion | 7 | 1848 | 3.16 | 0.89 | 2.95 | -0.87 | 0.79 |
|  | In my family we hardly ever lose our tempers | 8 | 1848 | 2.71 | 1.03 | 1.96 | -0.32 | 0.46 |
|  | There is strict punishment for anyone breaking the rules in my family | 9 | 1848 | 2.45 | 0.99 | 1.97 | 0.07 | 0.00 |
|  | We can do whatever we want in my family | 10 | 1848 | 1.67 | 0.83 | 3.82 | 1.18 | 0.70 |
|  | My family always does things together | 11 | 1848 | 2.96 | 0.92 | 2.49 | -0.57 | 0.76 |
|  | There are a lot of discussions in my family | 12 | 1848 | 2.03 | 0.86 | 2.64 | 0.54 | 0.40 |
|  | In my family we never hit each other | 13 | 1848 | 2.61 | 1.13 | 1.61 | -0.10 | 0.00 |
|  | “Work before play” is the rule in my family | 14 | 1848 | 2.83 | 0.98 | 2.12 | -0.39 | 0.57 |
|  | In my family we aren’t punished or told off when we do something wrong | 15 | 1848 | 2.07 | 0.96 | 2.40 | 0.58 | 0.46 |
|  | We really get along well with each other | 16 | 1848 | 3.14 | 0.91 | 2.94 | -0.87 | 0.75 |
|  | We don’t tell each other about our personal problems | 17 | 1848 | 2.20 | 1.01 | 2.07 | 0.40 | 0.00 |
|  | In my family we don’t often criticize each other | 18 | 1848 | 2.63 | 1.02 | 1.91 | -0.14 | 0.00 |
|  | Family members have strict ideas about what is right and what is wrong | 19 | 1848 | 2.98 | 0.96 | 2.46 | -0.65 | 0.51 |
|  | We come and go as we want to in my family | 20 | 1848 | 1.89 | 0.87 | 3.06 | 0.81 | 0.52 |
| **School bonding**  How much do you agree with the following descriptions of your school? Mark one box for each line. | The students in my class enjoy being together | 1 | 2043 | 2.00 | 0.87 | 2.65 | 0.58 | 0.76 |
|  | Most of the students in my class are kind and helpful | 2 | 2043 | 2.15 | 0.88 | 2.33 | 0.31 | 0.80 |
|  | Other students accept me as I am | 3 | 2043 | 1.94 | 0.88 | 2.80 | 0.70 | 0.76 |
|  | How I do in school matters a lot to me | 4 | 2043 | 1.83 | 0.91 | 2.89 | 0.88 | 0.43 |
|  | I have great respect for what my teachers tell me | 5 | 2043 | 1.76 | 0.80 | 3.37 | 0.90 | 0.57 |
| **Substance abuse index**  Have you ever had any of the following problems in the last 12 months?  Mark all that applies for each line. | Quarrel or argument | 1 | 2021 | 1.37 | 1.48 | 1.04 | 0.17 | 0.87 |
|  | Scuffle or fight | 2 | 2021 | 0.91 | 1.37 | 1.75 | 0.86 | 0.81 |
|  | Accident or injury | 3 | 2021 | 1.20 | 1.46 | 1.17 | 0.40 | 0.83 |
|  | Loss of money or other valuable items | 4 | 2021 | 0.62 | 1.20 | 3.17 | 1.46 | 0.80 |
|  | Damage to objects or clothing you owned | 5 | 2021 | 0.55 | 1.15 | 3.77 | 1.65 | 0.80 |
|  | Problems in your relationship with (your) parents | 6 | 2021 | 0.71 | 1.27 | 2.55 | 1.24 | 0.82 |
|  | Problems in your relationship with (your) friends | 7 | 2021 | 0.80 | 1.32 | 2.15 | 1.06 | 0.82 |
|  | Problems in your relationship with (your) teachers | 8 | 2021 | 0.48 | 1.09 | 4.48 | 1.86 | 0.77 |
|  | Performed poorly at school | 9 | 2021 | 0.85 | 1.34 | 1.95 | 0.97 | 0.81 |
|  | Victimized by robbery or theft | 10 | 2021 | 0.30 | 0.89 | 8.19 | 2.67 | 0.70 |
|  | Hospitalized or admitted to an emergency room | 11 | 2021 | 0.62 | 1.21 | 3.11 | 1.44 | 0.68 |
| **Decision making skills**  There are several possible ways to take decisions. How well do the following apply to you? Mark the answer that is closest to your opinion. | When I have decided to do something, I always carry it through | 1 | 2032 | 1.97 | 0.79 | 3.09 | 0.61 | 0.44 |
|  | I often make up my mind without thinking of the consequences | 2 | 2032 | 2.70 | 0.93 | 2.21 | -0.26 | 0.74 |
|  | I weigh up all the choices before I decide on something | 3 | 2032 | 2.00 | 0.88 | 2.65 | 0.60 | 0.83 |
|  | I often regret something that I had decided | 4 | 2032 | 2.25 | 0.94 | 2.12 | 0.22 | 0.44 |
|  | When I decide on something it doesn't matter what my friends think | 5 | 2032 | 2.63 | 1.02 | 1.90 | -0.17 | 0.47 |
| **Refusal skills**  Imagine yourself in each of the following situations. Some of them may be very familiar  to you, some others less, so that you may feel less secure in answering. It is enough you do your best. Mark the answer that is closest to your opinion. | You and your best friend are at a party where you meet new people, and you feel you really want to get to know them. Someone offers you to smoke hash together. Your friend accepts. Do you? | 1 | 2077 | 3.50 | 0.84 | 4.73 | -1.63 | 0.90 |
|  | You and the same friend are studying hard for an important test at school the day after. Both of you feel stressed and need to calm down. Your friend suggests a cigarette would help, and offers one. Do you accept? | 2 | 2077 | 3.54 | 0.81 | 5.08 | -1.72 | 0.93 |
|  | The day after, you both pass the test, and feel now it is time to celebrate. Have still some pocket-money left, and the liquor store is nearby. Would you buy some alcohol (beer, wine) to celebrate? | 3 | 2077 | 3.50 | 0.83 | 4.65 | -1.61 | 0.86 |
| **Self-esteem**  How much do you agree with the following descriptions of yourself?  Mark the answer that is closest to your opinion. | I feel that I have a number of good qualities | 1 | 1980 | 1.73 | 0.77 | 3.64 | 0.96 | 0.84 |
|  | I am able to do things as well as most other people | 2 | 1980 | 1.79 | 0.80 | 3.26 | 0.85 | 0.87 |
|  | At times I think I am no good at all | 3 | 1980 | 2.43 | 0.99 | 1.97 | 0.09 | 0.72 |
|  | Most boys and girls of my age are smarter than I am | 4 | 1980 | 2.64 | 0.91 | 2.35 | -0.34 | 0.72 |
|  | I am quite good at sports | 5 | 1980 | 2.04 | 0.97 | 2.36 | 0.60 | 0.64 |
|  | I feel very embarrassed when I have to say something in class | 6 | 1980 | 2.47 | 1.08 | 1.74 | 0.06 | 0.60 |
|  | My being happy is important to my parents | 7 | 1980 | 1.42 | 0.73 | 6.30 | 1.91 | 0.68 |
|  | I worry a lot about silly things | 8 | 1980 | 2.31 | 0.97 | 2.07 | 0.22 | 0.57 |
|  | I often feel nervous over nothing at al | 9 | 1980 | 2.62 | 1.00 | 1.99 | -0.21 | 0.00 |
|  | I have plenty of interests and hobbies | 10 | 1980 | 1.82 | 0.86 | 3.12 | 0.88 | 0.66 |
| **Poor problem solving skills**  Here are some statements about dealing with other people. Mark the answer that is closest to your  opinion. | When someone tries to make you feel small, you should do the same to them | 1 | 1997 | 2.69 | 1.00 | 2.05 | -0.31 | 0.81 |
|  | There is point in letting people know you're angry with them | 2 | 1997 | 2.39 | 0.95 | 2.10 | 0.11 | 0.54 |
|  | The only way to deal with bullies is to let them know who is in charge | 3 | 1997 | 2.60 | 1.00 | 1.96 | -0.17 | 0.75 |
|  | There are always ways of dealing with problems without having to fight | 4 | 1997 | 1.71 | 0.88 | 3.56 | 1.17 | 0.72 |
|  | It is much better to 'fly off the handle' than to explain things calmly | 5 | 1997 | 3.11 | 1.02 | 2.62 | -0.89 | 0.61 |
| **Assertiveness**  Imagine you would like to do the following things. How easy or difficult would you find  it? Mark one box for each line. | Say something nice to a friend | 1 | 2013 | 1.42 | 0.68 | 6.11 | 1.77 | 0.71 |
|  | Ask for a favor | 2 | 2013 | 1.71 | 0.76 | 3.39 | 0.89 | 0.75 |
|  | Show someone that I like him/her | 3 | 2013 | 2.74 | 1.08 | 1.86 | -0.37 | 0.00 |
|  | Say “no” when someone asks me to do something I do not want to | 4 | 2013 | 1.99 | 1.00 | 2.19 | 0.60 | 0.50 |
|  | Call for help when I have got problems | 5 | 2013 | 1.88 | 0.92 | 2.60 | 0.74 | 0.71 |
|  | Help someone who needs help | 6 | 2013 | 1.47 | 0.66 | 4.96 | 1.40 | 0.62 |
