# Supplementary material for: Validation of the European Drug Addiction Prevention Trial Questionnaire (EU-Dap) for substance use screening and to assess risk and protective factors among early adolescents in Chile
Source: PLoS One. 2021 Oct 11;16(10):e0258288. doi: 10.1371/journal.pone.0258288 (PMC8504767; doi:10.1371/journal.pone.0258288)
Supplement: S3 Table — (DOCX) [file pone.0258288.s005.docx]

S3 Table. Other risk and protective factors

| **Knowledge about substances** | **Items** |
| --- | --- |
| For each statement below, please mark whether you think it is correct or not by checking the appropriate box. (Yes, No, Don´t know) | Nicotine is the substance in cigarettes that causes lung cancer |
|  | One needs to smoke several cigarettes per day  during many years to become addicted |
|  | Women have lower tolerance to alcohol than men |
|  | It takes about half an hour to eliminate from the body  the amount of alcohol contained in a can of strong beer |
|  | Smoking marijuana does not cause physical dependence |
|  | High consumption of hash or marijuana decreases  the production of sexual hormones |
| **Tobacco use by family and friends** | **Items** |
| Do any of the following people smoke cigarettes? (Smokes daily, Smokes sometimes, Does not smoke, Don´t know, Don´t have or see this person) | Mother |
|  | Father |
|  | Best friend |
|  | Siblings |
| **Alcohol and drug use by siblings** | **Items** |
| Does any of your siblings? (Yes, No, Don´t know, Don´t have any siblings) | Drink alcoholic beverages (beer, wine, spirits) |
|  | Get drunk |
|  | Smoke marijuana or hashish (pot, grass) |
|  | Sniff substances (glue, petrol, paint thinner) |
|  | Take other drugs |
| **Parental permissiveness** | **Items** |
| If you wanted to smoke (or already do), do you think your father and mother would allow you to do so? (Would allow, Would not at home, Would not at all, Don´t know) | Father |
|  | Mother |
| If you wanted to drink alcohol (or already do), do you think your father and mother would allow you to do so? (Would allow, Would not at home, Would not at all, Don´t know) | Father |
|  | Mother |
| If you wanted to use marijuana (or already do), do you think your father and mother would allow you to do so? (Would allow, Would not at home, Would not at all, Don´t know) | Father |
|  | Mother |
| **Self-reported school performance** | **Items** |
| How did your grades compare with those of your classmates during the last school year? (Much better, Better, The same as most of them, Worse) | How did your grades compare with those of your classmates during the last school year. |
| **Positive academic expectations** | **Items** |
| In your opinion, will you have improved your grades at the end of this school year? (Yes, Probably yes, Probably no, No) | In your opinion, will you have improved your grades at the end of this school year? |
