# Supplementary material for: Validation of the European Drug Addiction Prevention Trial Questionnaire (EU-Dap) for substance use screening and to assess risk and protective factors among early adolescents in Chile
Source: PLoS One. 2021 Oct 11;16(10):e0258288. doi: 10.1371/journal.pone.0258288 (PMC8504767; doi:10.1371/journal.pone.0258288)
Supplement: S4 Table — (DOCX) [file pone.0258288.s006.docx]

# S4 Table. Subscales correlations.

|  | S1 | S2 | S3 | S4 | S5 | S6 | S7 | S8 | S9 | S10 | S11 | S12 | S13 | S14 | S15 | S16 | S17 | S18 | S19 | S20 | S21 |
| --- | --- | --- | --- | --- | --- | --- | --- | --- | --- | --- | --- | --- | --- | --- | --- | --- | --- | --- | --- | --- | --- |
| S1 | **1.00** | **0.10** | **0.72** | **0.07** | **0.66** | 0.04 | **0.25** | **-0.05** | -0.00 | **0.12** | **0.20** | **0.15** | **0.09** | **-0.08** | -0.01 | -0.03 | **-0.10** | **-0.05** | 0.05 | **-0.23** | **-0.10** |
| S2 | **0.10** | **1.00** | **0.13** | **0.76** | **0.09** | **0.71** | **-0.10** | **0.22** | **0.09** | 0.01 | -0.04 | -0.03 | -0.02 | **0.19** | 0.01 | **0.15** | **0.18** | **0.06** | -0.05 | **0.15** | 0.00 |
| S3 | **0.72** | **0.13** | **1.00** | **0.18** | **0.76** | **0.14** | **0.26** | -0.03 | 0.00 | **0.13** | **0.25** | **0.16** | **0.10** | -0.04 | -0.03 | -0.01 | **-0.07** | **-0.06** | **0.06** | **-0.24** | **-0.10** |
| S4 | **0.07** | **0.76** | **0.18** | **1.00** | **0.11** | **0.79** | **-0.13** | **0.24** | **0.12** | 0.02 | **-0.11** | -**0.05** | -0.04 | **0.19** | 0.03 | **0.17** | **0.19** | **0.06** | **-0.10** | **0.19** | -0.01 |
| S5 | **0.66** | **0.10** | **0.76** | **0.11** | **1.00** | **0.16** | **0.32** | -**0.05** | -0.03 | **0.14** | **0.26** | **0.15** | **0.10** | **-0.06** | -0.03 | -0.04 | **-0.16** | **-0.05** | **0.11** | **-0.27** | **-0.12** |
| S6 | 0.04 | **0.71** | **0.14** | **0.79** | **0.16** | **1.00** | **-0.14** | **0.28** | **0.13** | 0.02 | **-0.06** | -0.04 | -0.04 | **0.22** | 0.02 | **0.15** | **0.18** | **0.08** | **-0.08** | **0.17** | -0.02 |
| S7 | **0.25** | **-0.10** | **0.26** | **-0.13** | **0.32** | **-0.14** | **1.00** | **-0.08** | **-0.15** | **0.13** | **0.47** | **0.21** | **0.13** | **-0.19** | **-0.15** | **-0.14** | **-0.32** | **-0.14** | **0.15** | **-0.41** | **-0.06** |
| S8 | **-0.05** | **0.22** | -0.03 | **0.24** | **-0.05** | **0.28** | **-0.08** | **1.00** | **0.14** | -0.04 | **-0.12** | -0.01 | **-0.07** | **0.29** | 0.04 | **0.18** | **0.18** | **0.10** | -0.02 | **0.21** | -0.02 |
| S9 | -0.00 | **0.09** | 0.00 | **0.12** | -0.03 | **0.13** | **-0.15** | **0.14** | **1.00** | -0.02 | **-0.14** | **0.13** | **-0.08** | **0.41** | **0.24** | **0.42** | **0.08** | **0.44** | **0.09** | **0.14** | **-0.24** |
| S10 | **0.12** | 0.01 | **0.13** | 0.02 | **0.14** | 0.02 | **0.13** | -0.04 | -0.02 | **1.00** | **0.13** | **0.23** | 0.01 | **-0.06** | **-0.12** | -0.03 | **-0.09** | **-0.15** | **-0.06** | **-0.19** | **-0.32** |
| S11 | **0.20** | -0.04 | **0.25** | **-0.11** | **0.26** | **-0.06** | **0.47** | **-0.12** | **-0.14** | **0.13** | **1.00** | **0.13** | **0.19** | **-0.19** | **-0.14** | **-0.11** | **-0.20** | **-0.12** | **0.17** | **-0.42** | **-0.11** |
| S12 | **0.15** | -0.03 | **0.16** | **-0.05** | **0.15** | -0.04 | **0.21** | -0.01 | **0.13** | **0.23** | **0.13** | **1.00** | **0.07** | 0.01 | **-0.05** | -0.01 | **-0.10** | 0.04 | 0.02 | **-0.26** | **-0.27** |
| S13 | **0.09** | -0.02 | **0.10** | -0.04 | **0.10** | -0.04 | **0.13** | **-0.07** | **-0.08** | 0.01 | **0.20** | **0.07** | **1.00** | **-0.13** | -0.08 | **-0.07** | **-0.12** | **-0.05** | **0.11** | **-0.21** | **-0.06** |
| S14 | **-0.08** | **0.19** | -0.04 | **0.19** | **-0.06** | **0.22** | **-0.19** | **0.29** | **0.41** | **-0.06** | **-0.19** | 0.01 | **-0.13** | **1.00** | **0.21** | **0.36** | **0.12** | **0.38** | 0.02 | **0.27** | **-0.09** |
| S15 | -0.01 | 0.01 | -0.03 | 0.03 | -0.03 | 0.02 | **-0.15** | 0.04 | **0.24** | **-0.12** | **-0.14** | **-0.05** | **-0.08** | **0.21** | **1.00** | **0.19** | **0.15** | **0.19** | **0.09** | **0.17** | 0.01 |
| S16 | -0.03 | **0.15** | -0.01 | **0.17** | -0.04 | **0.15** | **-0.14** | **0.18** | **0.42** | -0.03 | **-0.11** | -0.01 | **-0.07** | **0.36** | **0.19** | **1.00** | **0.07** | **0.36** | **0.12** | **0.15** | **-0.14** |
| S17 | **-0.10** | **0.18** | **-0.07** | **0.19** | **-0.16** | **0.18** | **-0.32** | **0.18** | **0.08** | **-0.09** | **-0.20** | **-0.10** | **-0.12** | **0.12** | **0.15** | **0.07** | **1.00** | **0.09** | -0.03 | **0.24** | 0.04 |
| S18 | **-0.05** | **0.06** | **-0.06** | **0.06** | **-0.05** | **0.08** | **-0.14** | **0.10** | **0.44** | **-0.15** | **-0.12** | 0.04 | **-0.05** | **0.38** | **0.19** | **0.36** | **0.09** | **1.00** | 0.01 | **0.15** | **-0.12** |
| S19 | **0.14** | **-0.14** | **0.15** | **-0.18** | **0.19** | **-0.17** | **0.35** | **-0.11** | **-0.09** | **0.09** | **0.36** | **0.15** | **0.18** | **-0.17** | **-0.12** | **-0.15** | **-0.16** | **-0.13** | **1.00** | **-0.36** | **-0.08** |
| S20 | **-0.23** | **0.15** | **-0.24** | **0.19** | **-0.27** | **0.17** | **-0.41** | **0.21** | **0.14** | **-0.19** | **-0.42** | **-0.26** | **-0.21** | **0.27** | **0.17** | **0.15** | **0.24** | **0.15** | **-0.36** | **1.00** | **0.19** |
| S21 | **-0.10** | 0.00 | **-0.10** | -0.01 | **-0.12** | -0.02 | **-0.06** | -0.03 | **-0.24** | **-0.32** | **-0.11** | **-0.27** | **-0.06** | **-0.09** | 0.01 | **-0.14** | 0.04 | **-0.12** | 0.02 | **0.19** | **1.00** |

Note: correlations with p-values ≤ 0.05 are presented in bold. S1 = Positive beliefs about tobacco use subscale; S2 = Negative beliefs about tobacco use subscale; S3 = Positive beliefs about alcohol use subscale; S4 = Negative beliefs about alcohol use subscale; S5 = Positive beliefs about marijuana use subscale; S6 = Negative beliefs about marijuana use subscale; S7 = Positive attitudes towards drugs subscale; S8 = Negative attitudes towards drugs subscale; S9 = Positive Self-esteem subscale; S10 = Negative Self-esteem subscale; S11= Future substance use subscale; S12 = Poor problem-solving skills subscale; S13 = Substance abuse index subscale; S14 = Parental involvement subscale; S15 = Family functioning subscale; S16 = School bonding subscale; S17 = Risk perception subscale ; S18 = Assertiveness subscale; S19 = Normative beliefs subscale; S20 = Refusal skills; S21 = Decision-making skills subscale.
