# Supplementary material for: Validation of the European Drug Addiction Prevention Trial Questionnaire (EU-Dap) for substance use screening and to assess risk and protective factors among early adolescents in Chile
Source: PLoS One. 2021 Oct 11;16(10):e0258288. doi: 10.1371/journal.pone.0258288 (PMC8504767; doi:10.1371/journal.pone.0258288)
Supplement: S5 Table — (DOCX) [file pone.0258288.s007.docx]

S5 Table. Univariable associations of EU-Dap risk and protective and substance use.

| EU-Dap risk and protective factors | Tobacco use in the last 30 days | Alcohol use in the last 30 days | Drunk in the last 30 days | Binge drinking in the last 30 days Females (1) | Binge drinking in the last 30 days Males (1) | Marijuana use in the last 30 days | Marijuana use in the last 12 months |
| --- | --- | --- | --- | --- | --- | --- | --- |
|  | OR [95% CI]  p-value | OR [95% CI]  p-value | OR [95% CI]  p-value | OR [95% CI]  p-value | OR [95% CI]  p-value | OR [95% CI]  p-value | OR [95% CI]  p-value |
| Positive beliefs about tobacco use | 1.13** [1.05-1.21]  0.001 |  |  |  |  |  |  |
| Negative beliefs about tobacco use | 0.88** [0.82-0.95]  0.000 |  |  |  |  |  |  |
| Positive beliefs about alcohol use |  | 1.07** [1.04-1.11]  0.000 | 1.14** [1.07-1.22]  0.000 | 1.12** [1.05-1.19]  0.000 | 1.15** [1.07-1.23]  0.000 |  |  |
| Negative beliefs about alcohol use |  | 0.86** [0.83-0.89]  0.000 | 0.86** [0.81-0.93]  0.000 | 0.84** [0.79-0.90]  0.000 | 0.87** [0.81-0.94]  0.000 |  |  |
| Positive beliefs about marijuana use |  |  |  |  |  | 1.17** [1.10-1.26]  0.000 | 1.16** [1.09-1.22]  0.000 |
| Negative beliefs about marijuana use |  |  |  |  |  | 0.90** [0.86-0.94]  0.000 | 0.90** [0.87-0.93]  0.000 |
| Positive attitudes towards drugs |  |  |  |  |  | 1.56** [1.43-1.70]  0.000 | 1.45** [1.35-1.56]  0.000 |
| Negative attitudes towards drugs |  |  |  |  |  | 0.91** [0.87-0.95]  0.000 | 0.93** [0.89-0.96]  0.000 |
| Positive Self esteem | 0.91* [0.86-0.99]  0.017 | 0.95* [0.91-1.00]  0.039 | 0.91* [0.83-0.99]  0.033 | 0.87** [0.81-0.95]  0.001 | 0.92 [0.83-1.02]  0.122 | 0.96 [0.88-1.04]  0.321 | 0.95 [0.89-1.01]  0.099 |
| Negative Self esteem | 1.13* [1.04-1.23]  0.006 | 1.04 [0.99-1.09]  0.161 | 1.07 [0.96-1.20]  0.205 | 1.12* [1.02-1.24]  0.023 | 1.15* [1.01-1.30]  0.030 | 1.11* [1.01-1.22]  0.026 | 1.12* [1.03-1.21]  0.004 |
| *Removed item from Negative Self esteem* |  |  |  |  |  |  |  |
| Feel nervous over nothing | 1.16 [0.91-1.46]  0.228 | 1.05 [0.91-1.21]  0.507 | 1.04 [0.76-1.41]  0.819 | 1.11 [0.92-1.33]  0.265 | 1.11 [0.87-1.41]  0.405 | 1.67** [1.27-2.20]  0.000 | 1.36* [1.10-1.68]  0.004 |
| Future substance use | 1.24** [1.20-1.30]  0.000 | 1.21** [1.17-1.25]  0.000 | 1.23** [1.17-1.29]  0.000 | 1.29** [1.22-1.37]  0.000 | 1.19** [1.13-1.26]  0.000 | 1.25** [1.20-1.30]  0.000 | 1.24** [1.19-1.28]  0.000 |
| Poor problem-solving skills | 1.15** [1.06-1.24]  0.001 | 1.11** [1.06-1.17]  0.000 | 1.10 [0.99-1.28]  0.083 | 1.17** [1.07-1.29]  0.001 | 1.21* [1.07-1.37]  0.002 | 1.26** [1.15-1.38]  0.000 | 1.23** [1.14-1.32]  0.000 |
| Substance abuse index | 1.28** [1.13-1.44]  0.000 | 1.14* [1.02-1.28]  0.025 | 1.27* [1.08-1.49]  0.003 | 1.39** [1.15-1.68]  0.001 | 0.96 [0.63-1.45]  0.835 | 1.41** [1.26-1.58]  0.000 | 1.31** [1.18-1.47]  0.000 |
| Parental Involvement | 0.86** [0.81-0.92]  0.000 | 0.91** [0.87-0.95]  0.000 | 0.89* [0.82-0.97]  0.008 | 0.87** [0.80-0.95]  0.001 | 0.95 [0.85-1.06]  0.387 | 0.86** [0.80-0.92]  0.000 | 0.86** [0.81-0.91]  0.000 |
| Family functioning | 0.94** [0.91-0.97]  0.000 | 0.97* [0.95-1.00]  0.019 | 0.95* [0.91-0.99]  0.023 | 0.96* [0.92-0.99]  0.032 | 0.96 [0.91-1.01]  0.090 | 0.93** [0.90-0.97]  0.000 | 0.94** [0.91-0.97]  0.000 |
| *Removed item from Family functioning* |  |  |  |  |  |  |  |
| Punishment when breaking the rules | 0.90 [0.71-1.15]  0.402 | 0.81* [0.70-93.8]  0.005 | 0.65* [0.47-0.90]  0.010 | 0.75 [0.56-1.00]  0.051 | 0.81 [0.58-1.14]  0.229 | 0.77 [0.58-1.01]  0.060 | 0.86 [0.69-1.06]  0.156 |
| Never hit each other | 0.75* [0.60-0.92]  0.006 | 0.99 [0.87-1.12]  0.822 | 0.83 [0.63-1.08]  0.161 | 0.77* [0.61-0.99]  0.042 | 0.74 [0.54-1.01]  0.058 | 0.66** [0.52-0.85]  0.001 | 0.73** [0.61-0.89]  0.001 |
| Tell each other problems | 0.91 [0.73-1.15]  0.444 | 0.97 [0.84-1.11]  0.637 | 0.96 [0.72-1.29]  0.796 | 0.75* [0.58-0.98]  0.032 | 1.13 [0.78-1.63]  0.511 | 1.08 [0.82-1.41]  0.594 | 0.98 [0.79-1.20]  0.811 |
| Don’t often criticize each other | 0.72* [0.57-0.91]  0.006 | 0.91 [0.79-1.05]  0.217 | 0.76 [0.56-1.02]  0.067 | 0.85 [0.65-1.11]  0.224 | 0.85 [0.60-1.20]  0.355 | 0.85 [0.65-1.11]  0.245 | 1.08 [0.87-1.33]  0.477 |
| School bonding | 0.85** [0.80-0.91]  0.000 | 0.90** [0.86-0.94]  0.000 | 0.86** [0.79-0.94]  0.001 | 0.84** [0.77-0.92]  0.000 | 0.93 [0.84-1.04]  0.197 | 0.96 [0.88-1.04]  0.339 | 0.89** [0.83-0.95]  0.000 |
| Risk Perception | 0.90* [0.82-0.98]  0.020 | 0.95 [0.90-1.02]  0.143 | 1.01 [0.85-1.19]  0.929 | 0.91 [0.81-1.02]  0.096 | 0.92 [0.80-1.06]  0.258 | 0.80** [0.74-0.87]  0.000 | 0.82** [0.77-0.88]  0.000 |
| Assertiveness | 0.89* [0.82-0.96]  0.002 | 0.95 [0.91-1.00]  0.071 | 0.90* [0.81-0.99]  0.038 | 0.95 [0.86-1.05]  0.323 | 0.91 [0.81-1.02]  0.122 | 0.93 [0.85-1.02]  0.132 | 0.94 [0.87-1.00]  0.912 |
| *Removed item from Assertiveness* |  |  |  |  |  |  |  |
| Show someone that likes him/her | 1.57** [1.26-1.95]  0.000 | 1.26** [1.10-1.44]  0.001 | 1.29 [0.98-1.71]  0.073 | 1.71** [1.33-2.19]  0.000 | 1.56* [1.10-2.20]  0.012 | 1.60** [1.24-2.05]  0.000 | 1.63** [1.34-1.98]  0.000 |
| Normative beliefs | 1.56** [1.41-1.72]  0.000 | 1.53** [1.40-1.68]  0.000 | 1.50** [1.33-1.68]  0.000 | 1.52** [1.32-1.76]  0.000 | 1.52** [1.33-1.74]  0.000 | 1.55** [1.39-1.72]  0.000 | 1.64** [1.48-1.82]  0.000 |
| *Removed items from Normative beliefs* |  |  |  |  |  |  |  |
| How many of them like school? | 0.93 [0.75-1.16]  0.542 | 0.94 [0.82-1.06]  0.310 | 1.08 [0.82-1.42]  0.585 | 0.84 [0.65-1.08]  0.171 | 0.88 [0.66-1.19]  0.413 | 1.00 [0.78-1.27]  0.985 | 0.78* [0.63-0.95]  0.016 |
| How many of them do well at school? | 0.68* [0.51-0.90]  0.007 | 0.79* [0.67-0.93]  0.004 | 0.90 [0.64-1.28]  0.561 | 0.67* [0.49-0.91]  0.012 | 0.58* [0.38-0.89]  0.013 | 0.73 [0.52-1.01]  0.056 | 0.59** [0.45-0.78]  0.000 |
| Refusal skills | 0.72** [0.67-0.78]  0.000 | 0.74** [0.70-0.78]  0.000 | 0.72** [0.65-0.79]  0.000 | 0.68** [0.61-0.74]  0.000 | 0.74** [0.66-0.83]  0.000 | 0.69** [0.63-0.75]  0.000 | 0.70** [0.65-0.75]  0.000 |
| Decision-making skill | 0.83** [0.76-0.91]  0.000 | 0.95 [0.90-1.00]  0.093 | 0.90 [0.80-1.01]  0.074 | 0.88* [0.79-0.97]  0.015 | 0.87* [0.77-0.99]  0.038 | 0.79** [0.72-0.87]  0.000 | 0.81** [0.75-0.88]  0.000 |
| Other items included in the questionnaire |  |  |  |  |  |  |  |
| Self-reported school performance |  |  |  |  |  |  |  |
| Worse than classmates (reference) | 1 | 1 | 1 | 1 | 1 | 1 | 1 |
| Same as classmates | 0.50* [0.27-0.94]  0.031 | 0.55* [0.35-0.86]  0.008 | 0.60 [0.26-1.41]  0.243 | 0.32* [0.16-0.66]  0.002 | 0.80 [0.28-2.31]  0.677 | 0.70 [0.33-1.49]  0.360 | 0.62 [0.35-1.10]  0.102 |
| Better or much better than classmates | 0.30** [0.16-0.57]  0.000 | 0.53* [0.35-0.81]  0.003 | 0.40* [0.17-0.95]  0.037 | 0.21** [0.10-0.44]  0.000 | 0.71 [0.26-1.95]  0.503 | 0.39* [0.18-0.85]  0.018 | 0.36** [0.20-0.65]  0.001 |
| Positive academic expectations | 0.84 [0.64-1.12]  0.233 | 0.73** [0.61-0.86]  0.000 | 0.66* [0.47-0.93]  0.017 | 0.85 [0.61-1.19]  0.348 | 0.81 [0.55-1.21]  0.304 | 0.86 [0.63-1.19]  0.367 | 0.80 [0.63-1.02]  0.078 |
| Parental permissiveness |  |  |  |  |  |  |  |
| Father´s permissiveness: Tobacco | 3.50** [2.37-5.15]  0.000 | 1.52* [1.09-2.11]  0.014 | 2.30* [1.32-4.04]  0.004 | 2.50** [1.43-4.34]  0.001 | 0.90 [0.31-2.58]  0.844 | 3.34** [2.12-5.27]  0.000 | 3.38** [2.32-4.91]  0.000 |
| Mother´s permissiveness: Tobacco | 4.30** [2.97-6.22]  0.000 | 1.65* [1.19-2.29]  0.003 | 2.54** [1.48-4.36]  0.001 | 3.03** [1.79-5.13]  0.000 | 1.13 [0.46-2.79]  0.784 | 3.20** [2.04-5.02]  0.000 | 3.55** [2.47-5.10]  0.000 |
| Father´s permissiveness: Alcohol | 1.96** [1.34-2.85]  0.000 | 2.77** [2.21-3.46]  0.000 | 1.73* [1.06-2.81]  0.028 | 2.04* [1.28-3.27]  0.003 | 2.51** [1.48-4.27]  0.001 | 2.58** [1.73-3.85]  0.000 | 2.81** [2.02-3.90]  0.000 |
| Mother´s permissiveness: Alcohol | 2.25** [1.57-3.21]  0.000 | 2.88** [2.30-3.60]  0.000 | 2.80** [1.79-4.38]  0.000 | 3.77** [2.53-5.63]  0.000 | 2.11* [1.26-3.54]  0.005 | 3.46** [2.35-5.09]  0.000 | 3.12** [2.31-4.22]  0.000 |
| Father´s permissiveness: Marijuana | 3.29** [2.22-4.88]  0.000 | 1.69* [1.20-2.38]  0.003 | 2.38* [1.34-4.23]  0.003 | 2.37* [1.38-4.06]  0.002 | 1.98 [0.93-4.19]  0.076 | 3.83** [2.40-6.09]  0.000 | 4.62** [3.21-6.65]  0.000 |
| Mother´s permissiveness: Marijuana | 3.21** [2.18-4.73]  0.000 | 1.52* [1.08-2.13]  0.016 | 2.83** [1.70-4.71]  0.000 | 2.43** [1.45-4.06]  0.001 | 1.29 [0.53-3.13]  0.570 | 4.16** [2.74-6.33]  0.000 | 4.08** [2.87-5.79]  0.000 |
| Family and friends’ tobacco use |  |  |  |  |  |  |  |
| Mother | 2.25** [1.66-3.04]  0.000 | 1.43** [1.18-1.73]  0.000 | 2.05** [1.40-3.02]  0.000 | 1.54* [1.09-2.19]  0.015 | 1.46 [0.94-2.28]  0.094 | 1.65* [1.17-2.33]  0.005 | 1.82** [1.39-2.38]  0.000 |
| Father | 1.71** [1.27-2.30]  0.000 | 1.25* [1.02-1.52]  0.030 | 1.74* [1.18-2.57]  0.005 | 1.34 [0.93-1.94]  0.116 | 1.23 [0.76-2.0]  0.396 | 1.84** [1.31-2.59]  0.000 | 1.79** [1.36-2.34]  0.000 |
| Siblings | 2.13** [1.52-2.97]  0.000 | 2.03** [1.64-2.52]  0.000 | 2.27** [1.47-3.49]  0.000 | 2.18** [1.46-3.26]  0.000 | 1.23 [0.68-2.22]  0.492 | 2.27** [1.56-3.32]  0.000 | 1.99** [1.45-2.71]  0.000 |
| Best friends | 6.13** [4.29-8.76]  0.000 | 2.79** [2.11-3.68]  0.000 | 3.48** [2.13-5.68]  0.000 | 3.89** [2.45-6.17]  0.000 | 2.84** [1.60-5.02]  0.000 | 5.12** [3.40-7.72]  0.000 | 4.53** [3.226.36]  0.000 |
| Siblings´ Alcohol and Drugs use |  |  |  |  |  |  |  |
| Alcohol | 1.58 [0.94-2.67]  0.085 | 2.99** [2.20-4.08]  0.000 | 2.97* [1.51-5.85]  0.002 | 2.85** [1.56-5.20]  0.001 | 3.54** [1.72-7.26]  0.001 | 1.01 [0.52-1.97]  0.975 | 1.44 [0.90-2.30]  0.130 |
| Drunkenness | 2.79* [1.48-5.26]  0.002 | 3.78** [2.56-5.59]  0.000 | 2.16 [0.88-5.31]  0.093 | 4.69** [2.39-9.21]  0.000 | 2.66* [1.05-6.69]  0.038 | 1.40 [0.59-3.36]  0.443 | 1.92* [1.04-3.55]  0.038 |
| Marijuana | 2.99** [1.58-5.65]  0.001 | 3.05* *[2.03-4.58]  0.000 | 3.54* [1.56-8.03]  0.003 | 2.31* [1.03-5.16]  0.041 | 3.46* [1.51-7.91]  0.003 | 5.14** [2.72-9.72]  0.000 | 4.98** [2.99-8.29]  0.000 |
| Inhalants | 13.13** [4.93-34.92]  0.000 | 2.25 [0.75-6.73]  0.146 | 2.69 [0.35-20.59]  0.341 | 4.70 [0.98-22.47]  0.053 | 1.00 | 13.54** [4.75-38.54]  0.000 | 9.55** [3.63-25.17]  0.000 |
| Other drugs | 2.54 [0.76-8.49]  0.129 | 3.27** [1.58-6.77]  0.001 | 2.79 [0.65-12.06}  0.169 | 1.00 | 4.24* [1.20-14.93]  0.025 | 3.14 [0.94-10.57]  0.064 | 3.94* [1.61-9.64]  0.003 |
| Knowledge about substances |  |  |  |  |  |  |  |
| Knowledge about tobacco | 0.76 [0.49-1.19]  0.236 | 1.07 [0.80-1.44]  0.615 | 1.03 [0.56-1.91]  0.913 | 0.69 [0.38-1.28]  0.243 | 1.46 [0.74-2.88]  0.270 | 0.74 [0.44-1.23]  0.245 | 0.64* [0.42-0.97]  0.036 |
| Knowledge about alcohol | 0.71 [0.35-1.43]  0.337 | 1.45 [0.87-2.40]  0.155 | 0.63 [0-23-1.69]  0.359 | 0.42 [0.17-1.06]  0.067 | 2.20 [0.80-6.03]  0.125 | 0.93 [0.44-1.94]  0.847 | 1.32 [0.66-2.62]  0.428 |
| Knowledge about marihuana | 0.66 [0.36-1.23]  0.191 | 0.98 [0.61-1.57]  0.933 | 1.09 [0.47-2.54]  0.841 | 1.56 [0.58-4.22]  0.380 | 0.17* [0.05-0.51]  0.002 | 0.51 [0.26-1.02]  0.056 | 0.48* [0.27-0.85]  0.012 |

Note: Empty cells indicate that the variables did not enter into the model. *p≤0.05 and **p≤0.001. (1) For binge drinking, there are two different definitions according to gender. In the case of females, the variable is defined using 4 or more drinks on the same occasion (2 hours); while in males, the variable is defined as 5 or more drinks on the same occasion (2 hours).
